# New Insights into Plagiogrammaceae (Bacillariophyta) Based on Multigene Phylogenies and Morphological Characteristics with the Description of a New Genus and Three New Species

**DOI:** 10.1371/journal.pone.0139300

**Published:** 2015-10-14

**Authors:** Chun L. Li, Matt P. Ashworth, Andrzej Witkowski, Przemysław Dąbek, Linda K. Medlin, Wiebe H. C. F. Kooistra, Shinya Sato, Izabela Zgłobicka, Krzysztof J. Kurzydłowski, Edward C. Theriot, Jamal S. M. Sabir, Mohammad A. Khiyami, Mohammed H. Z. Mutwakil, Meshaal J. Sabir, Njud S. Alharbi, Nahid H. Hajarah, Song Qing, Robert K. Jansen

**Affiliations:** 1 Palaeoceanology Unit, Faculty of Geosciences, University of Szczecin, Mickiewicza 18, PL-70-383, Szczecin, Poland; 2 Department of Integrative Biology, University of Texas at Austin, Austin, Texas, United States of America; 3 Marine Biological Association of the UK, The Citadel, Plymouth, United Kingdom PL1 2 PB; 4 Department of Integrative Marine Ecology, Stazione Zoologica Anton Dohrn, Villa Comunale, 80121 Napoli, Italy; 5 Faculty of Marine Bioscience, Fukui Prefectural University, Fukui, Japan; 6 Faculty of Materials Science and Engineering, Warsaw University of Technology, Warsaw, Poland; 7 Texas Memorial Museum, University of Texas at Austin, Austin, Texas, United States of America; 8 Biotechnology Research Group, Department of Biological Sciences, Faculty of Science, King Abdulaziz University, Jeddah 21589, Saudi Arabia; 9 King Abdulaziz City for Science and Technology, Riyadh, Saudi Arabia; 10 Yantai Institute of Coastal Zone Research, Chinese Academy of Sciences, Yantai, China; Field Museum of Natural History, UNITED STATES

## Abstract

Plagiogrammaceae, a poorly described family of diatoms, are common inhabitants of the shallow marine littoral zone, occurring either in the sediments or as epiphytes. Previous molecular phylogenies of the Plagiogrammaceae were inferred but included only up to six genera: *Plagiogramma*, *Dimeregramma*, *Neofragilaria*, *Talaroneis*, *Psammogramma* and *Psammoneis*. In this paper, we describe a new plagiogrammoid genus, *Orizaformis*, obtained from Bohai Sea (China) and present molecular phylogenies of the family based on three and four genes (nuclear-encoded large and small subunit ribosomal RNAs and chloroplast-encoded *rbc*L and *psb*C). Also included in the new phylogenies is *Glyphodesmis*. The phylogenies suggest that the Plagiogrammaceae is composed of two major clades: one consisting of *Talaroneis*, *Orizaformis* and *Psammoneis*, and the second of *Glyphodesmis*, *Psammogramma*, *Neofragilaria*, *Dimeregramma* and *Plagiogramma*. In addition, we describe three new species within established genera: *Psammoneis obaidii*, which was collected from the Red Sea, Saudi Arabia; and *Neofragilaria stilus* and *Talaroneis biacutifrons* from the Mozambique Channel, Indian Ocean, and illustrate two new combination taxa: *Neofragilaria anomala* and *Neofragilaria lineata*. Our observations suggest that the biodiversity of the family is strongly needed to be researched, and the phylogenetic analyses provide a useful framework for future studies of Plagiogrammaceae.

## Introduction

The araphid diatom family Plagiogrammaceae was established by De Toni in 1890, and was often confused with other small araphid diatoms because of its broadly defined characters. Seven genera are currently included in the family: *Plagiogramma* Greville, *Dimeregramma* Ralfs, *Glyphodesmis* Greville, *Neofragilaria* Desikachary, Prasad & Prema, *Psammogramma* Sato & Medlin, *Psammoneis* Sato, Kooistra & Medlin and *Talaroneis* Kooistra & De Stefano, which was proposed to replace the invalidly published *Dimeregrammopsis* Ricard. A recently proposed circumscription of this family by Sato et al. [1] defined the group thus: (1) elongated valves with parallel striae, oriented perpendicular to the apical axis; (2) a visible sternum in most genera; (3) apical pore fields on valves; (4) absence of rimoportula; and (5) presence of occluded areolae. However, these morphological characteristics of Plagiogrammaceae are not unique among diatoms. Many araphid diatoms have parallel striae and a distinct sternum; many araphid diatoms (and even some centric diatoms, particularly in the Biddulphiaceae and Eupodiscaceae) have apical pore fields; other small araphid pennates (such as *Staurosira* species) and centrics (*Corethron* and *Leptocylindrus*) lack rimoportula; and occluded pores are found in most diatoms, albeit in a variety of siliceous structures. Additionally, these characters are not universally shared by all genera within the Plagiogrammaceae. *Psammoneis* is the only plagiogrammoid genus in which siliceous areolae occlusions have not been observed thus far, only occasionally remnants of areolar occlusion have been observed [1]. Also, little is known about the biology of these taxa. All we know about their ecology is that they are abundant in the marine littoral zone [1–3].

Perhaps because of morphological ambiguity, the taxonomic position of the Plagiogrammaceae has been controversial. Initially, the family was placed in the araphid diatoms [2,4,5] due to a distinct sternum and a valve outline typical for pennate diatoms. Round & Crawford [6] later transferred Plagiogrammaceae into the subclass Biddulphiophycidae Round & Crawford, the order Triceratiales Round & Crawford, and the family Triceratiaceae. The reason for this was not clearly stated but was likely related to the presence of elaborate apical pore fields resembling ocelli and pseudocelli, the latter characterizing the Biddulphiophycidae [6]. Kooistra et al. [7] revised the taxonomic position of the Plagiogrammaceae suggesting once again that it belonged in the araphid pennates because an analysis of nuclear-encoded ribosomal small subunit (SSU) DNA sequences from a number of diatom lineages placed the plagiogrammacean *Talaroneis posidoniae* Kooistra & de Stefano sister to araphid pennates *Asterionellopsis glacialis* (Castracane) Round, *Asteroplanus karianus* Gardner & Crawford, and *Rhaphoneis* cf. *belgica* [7]. Several recent molecular phylogenies of diatoms [1,3,8–10] indicated that the Plagiogrammaceae were part of araphid diatoms and positioned in a clade containing some fragilariacean and rhaphoneidacean diatoms at the base of araphid pennate diatoms.

Despite these previous studies, evolutionary relationships among the genera in this family are still unresolved. The phylogeny inferred from LSU rDNA demonstrated that *Talaroneis* and *Psammogramma* formed a clade sister to the clade containing *Dimeregramma*, *Neofragilaria* and *Plagiogramma*, with the latter two being sister taxa [1]. An 18S rDNA molecular phylogeny revealed that *Talaroneis* diverged first in the Plagiogrammaceae clade, followed by a clade with *Neofragilaria* and *Plagiogramma* and then by a clade of *Dimeregramma* and *Psammoneis* [3]. A phylogeny based on three genes (18S rDNA, *rbc*L and *psb*C) recovered *Dimeregramma* and *Plagiogramma* as a clade sister to *Psammoneis* [10]. There is little consistency regarding the phylogenetic relationships among the genera within Plagiogrammaceae. This is likely due to sampling bias, because only one of the previous studies used multiple genes, and most genera were only represented by a single taxon, or were not included.

Here we expanded twenty-six clones of plagiogrammoid taxa studied morphologically and molecularly. Species belonging to the Plagiogrammaceae have been studied from samples collected in coastal regions worldwide and successfully grown as clonal cultures. To resolve evolutionary relationships among the lineages of the Plagiogrammaceae, we assembled a multigene dataset with all Plagiogrammacean genera (SSU, LSU, *rbc*L and *psb*C for all except *Glyphodesmis*, for which we only have *rbc*L and *psb*C data). The resulting phylogenetic analyses are the most complete sampling of the genetic relationships among lineages of plagiogrammoid genera to date. In addition, our study examines the morphology of the valves using scanning electron microscopy (SEM) and transmission electron microscopy (TEM) to search for unambiguous diagnostic characters for the genera of the Plagiogrammaceae. This is the first attempt to examine all the known genera of the Plagiogrammaceae from a morphological and molecular standpoint, which will be valuable for future surveys of small, marine araphid diatoms (*e*.*g*., *Opephora*, *Pseudostaurosira*, *Nanofrustulum*) because there will finally be a phylogenetically based morphological and molecular circumscription of plagiogrammacean taxa. We also attempt to analyze how frustule morphology has evolved in the Plagiogrammaceae, which is key to understanding the evolution of araphid pennates because Plagiogrammaceae are one of the earliest-diverging lineages of pennate diatoms. Finally, we contribute to the knowledge of biodiversity of Plagiogrammaceae, adding a new genus, *Orizaformis* with one new species, *O*. *holarctica* from Bohai (China) and three new species: *Psammoneis obaidii* from the Red Sea, *Neofragilaria stilus* and *Talaroneis biacutifrons* both from the Indian Ocean. Molecular analyses support our interpretation and we include *Orizaformis* in the Plagiogrammaceae.

## Results

### Phylogenetic analyses

**Fig 1:**
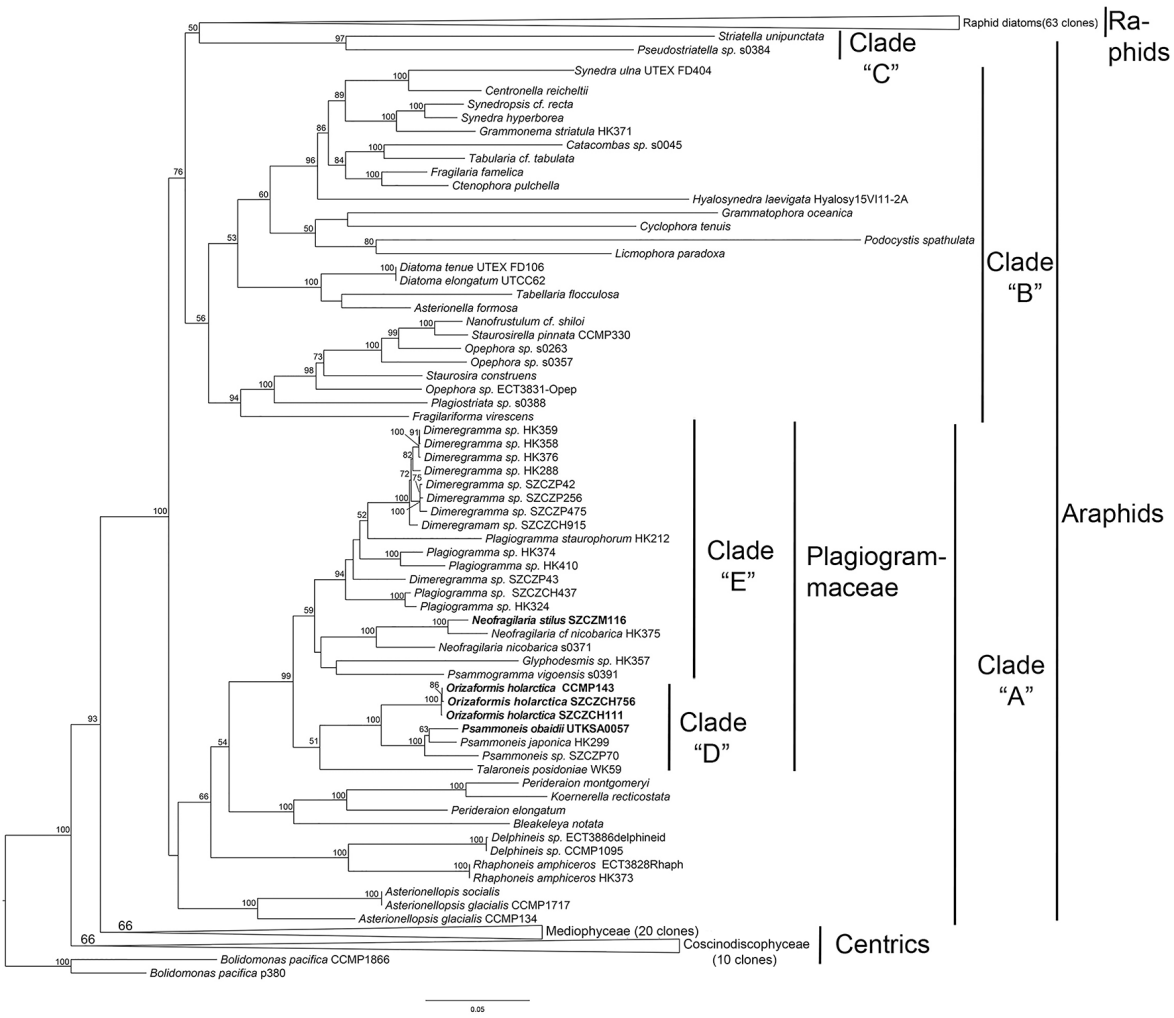
Maximum likelihood phylogeny of 161 diatoms (with bootstrap values at nodes) was inferred from a concatenated alignment of SSU rRNA, *rbc*L and *psb*C markers. Taxa (*Neofragilaria stilus*, *Orizaformis holarctica* and *Psammoneis obaidii*) in bold are newly described species. Support values less than 50% were omitted. Two *Bolidomona spacifica* species were used as outgroups. Centric and raphid clades were collapsed.

The phylogeny of the diatoms was inferred with Maximum Likelihood (ML) analysis from 161 taxa with a three-gene dataset (SSU, *rbc*L and *psb*C), using two *Bolidomonas pacifica* strains as the outgroups. Our analysis recovered centrics and araphids as paraphyletic, and raphids as monophyletic (Fig 1). Centrics were divided into clades corresponding to the Mediophyceae and Coscinodiscophyceae [11], with the former as sister to pennates. Araphids were split into three clades: araphid clade “A”, consisting of plagiogrammacean diatoms, *Perideraion elongatum* Jordan, Arai & Lobban, *Perideraion montgomeryi* Lobban, Jordan & Ashworth, *Koernerella recticostata* (Körner) Ashworth, Lobban & Theriot, *Bleakeleya notata* (Grunow) Round, Rhaphoneidaceae and *Asterionellopsis spp*.; then the core araphid clade “B”, which was sister to a clade containing raphid diatoms plus araphid clade “C”, composed of *Striatella unipunctata* (Lyngbye) C.Agardh and *Pseudostriatella* Sato, D.G. Mann & Medlin.

The phylogenetic tree (Fig 1) indicated that the newly described species (except *Talaroneis biacutifrons*, which lacks molecular data) were positioned in Plagiogrammaceae, which was monophyletic (bootstrap value [bv] = 99%) and sister to a clade containing *Perideraion*, *Koernerella* and *Bleakeleya* (bv = 54%). This assemblage was sister to the *Rhaphoneis* spp. and *Delphineis* spp. (bv = 66%), and then to an unresolved trichotomy with *Asterionellopsis* spp. Plagiogrammaceae was further divided into two subclades; clade “D”, comprised of *Orizaformis* (this new genus would be described in the taxonomic revisions section), *Psammoneis* and *Talaroneis* (bv = 51%), with *Talaroneis posidoniae* sister to a well-supported clade *Orizaformis*+*Psammoneis* (bv = 100%); clade “E” (bv = 59%) including the remaining taxa split into two subclades. One is *Glyphodesmis*+*Psammogramma+Neofragilaria* clade, where the clade *Glyphodesmis*+*Psammogramma* (bv < 50%) were sister to a monophyletic *Neofragilaria* group (bv = 100%), in which the newly described *Neofragilaria stilus* was sister to *Neofragilaria* cf. *nicobarica* (bv = 100%). *Dimeregramma* and *Plagiogramma* formed the second subclade (bv = 94%), but neither genus was monophyletic because one clone of *Dimeregramma sp*. (SZCZP43) was nested within a *Plagiogramma* clade of (HK374, HK410), but with very low support.

**Fig 2:**
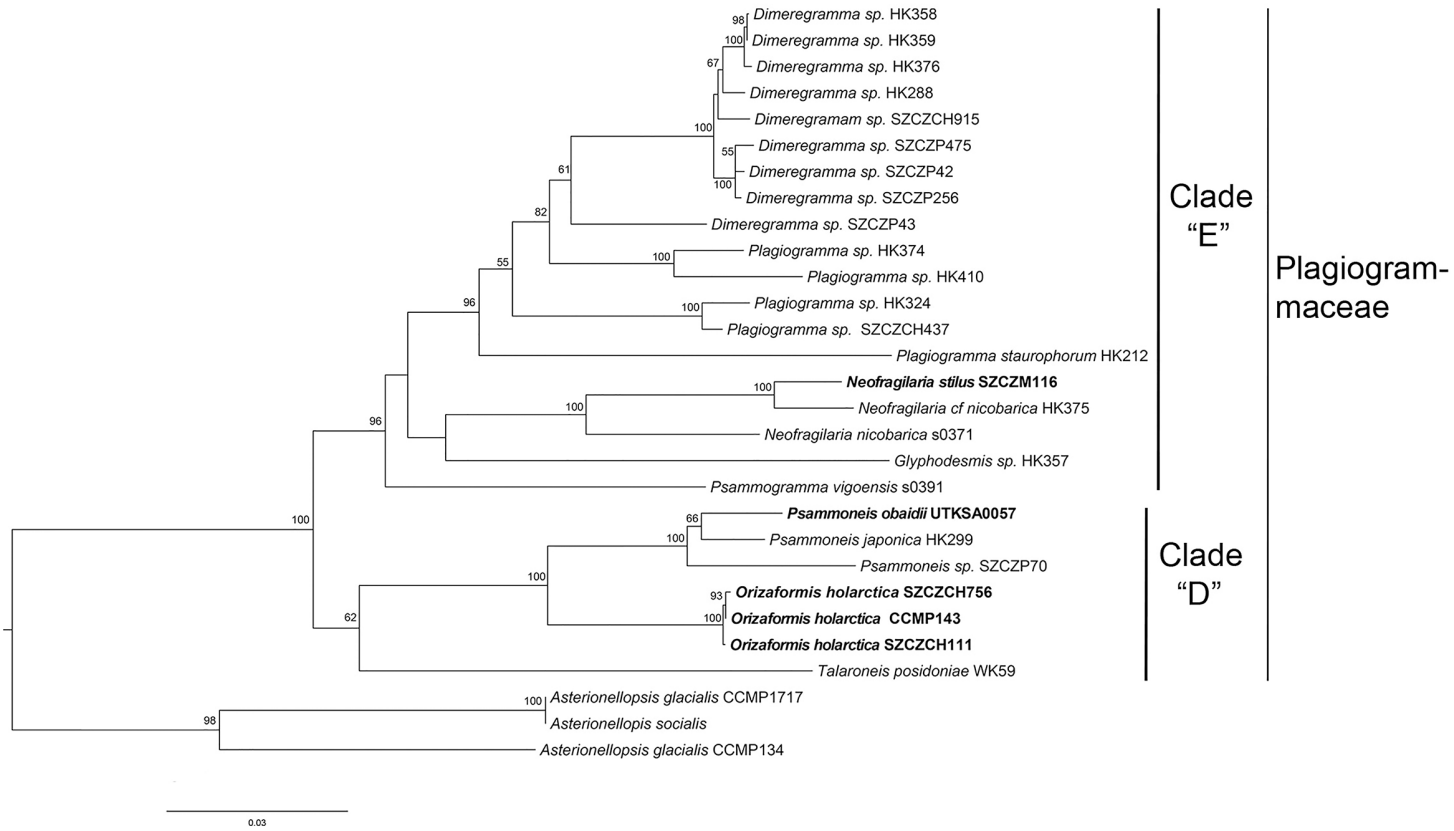
Maximum likelihood phylogeny of 26 strains of Plagiogrammacean diatoms (with bootstrap values at nodes) was inferred from four markers (LSU, SSU rDNA, *rbc*L and *psb*C). Taxa (*Neofragilaria stilus*, *Orizaformis holarctica* and *Psammoneis obaidii*) in bold are newly described species. Support values lower than 50% were omitted. The outgroup *Asterionella formosa* was removed from the tree.

We performed another ML analysis with a four-gene (SSU, *rbc*L, *psb*C and LSU) dataset, focusing only on the Plagiogrammaceae plus *Asterionellopsis* spp. using *Asterionella formosa* as an outgroup. This analysis yielded a similar tree topology as the three-gene tree, in which Plagiogrammaceae was divided into clade “D” and clade “E” (Fig 2). However, in clade “E”, a clade (bv = 96%) containing a monophyletic *Dimeregramma* (bv = 61%) and a grade clade of *Plagiogramma* were recovered, and *Psammogramma vigoensis* was sister to the clade that contains *Dimeregramma*, *Plagiogramma*, *Neofragilaria* and *Glyphodesmis* (bv = 96%), rather than sister to *Glyphodesmis* as in three-gene tree (Fig 1). The support values of a few branches were different between four-gene and three-gene analyses. The Plagiogrammaceae clade is well supported in both analyses (100 vs 99%). The clade “D” received a slightly better support in four-gene analysis (62 vs 51%). And clade “E” is much better supported in four-gene three (96 vs 59%).

When we constrained trees (three-gene and four-gene datasets) to a topology with both *Plagiogramma* and *Dimeregramma* monophyletic, the Shimodaira-Hasegawa (SH) tests (Table 1) showed that they were not significantly different between constrained trees (S1 and S2 Figs) and unconstrained trees (Figs 1 and 2) for both datasets.

**Table 1:** Shimodaira-Hasegawa (SH) test for the topologically unconstrained trees (Figs 1 and 2) and constrained trees (S1 and S2 Figs) in three-gene and four-gene analyses. Significance was set as alpha = 0.05. The difference in likelihood (ΔlnL) was calculated by subtracting the log likelihood score between the unconstrained and constrained best tree.

Best trees	-lnL	ΔlnL	SD	Significantly Worse
Unconstrained three-gene tree	-105129.984			
Constrained three-gene tree	-105139.748	-9.763	19.416	No
Unconstrained four-gene tree	-18924.790			
Constrained four-gene tree	-18939.503	-14.713	8.845	No

### Taxonomic revisions

#### Description of the genus Orizaformis

*Orizaformis Witkowski*, *Chunlian Li & Ashworth gen*. *nov*.:

Description: Two Chloroplasts per cell, plate-like against the girdle region and slightly overlapping onto the valve face. Frustules are rectangular in girdle view, solitary or forming zigzag colonies. Girdle relatively broad, composed of numerous, perforated bands under eletron microscope (EM). Valves linear sometimes expanded in the middle with obtusely rounded apices. Morphological details in LM barely recognizeable, transapical striae very dense, sternum not recognizable. In SEM sternum narrow, linear and the transapical striae parallel throughout, regularly spaced and composed of small oblong areolae. No areolae occlusions have been observed thus far. At both apices relatively large and well-developed pore fields composed of apically oriented rows of puncta occur. Rimoportulae are absent.

Type species: *Orizaformis holarctica* Witkowski, Chunlian Li & Ashworth sp. nov.

Etymology: the name of the new genus is derived from “Oriza”, the Latin name of rice. “*Orizaformis*” refers to the resemblance of the valves of the new genus to a rice grain.

Habitats: recorded so far only in marine environment of Bohai Sea in China, the East coast of Hokkaido Island, Japan (West Pacific) and the shore of Moss Landing, CA, USA.

**Fig 3:**
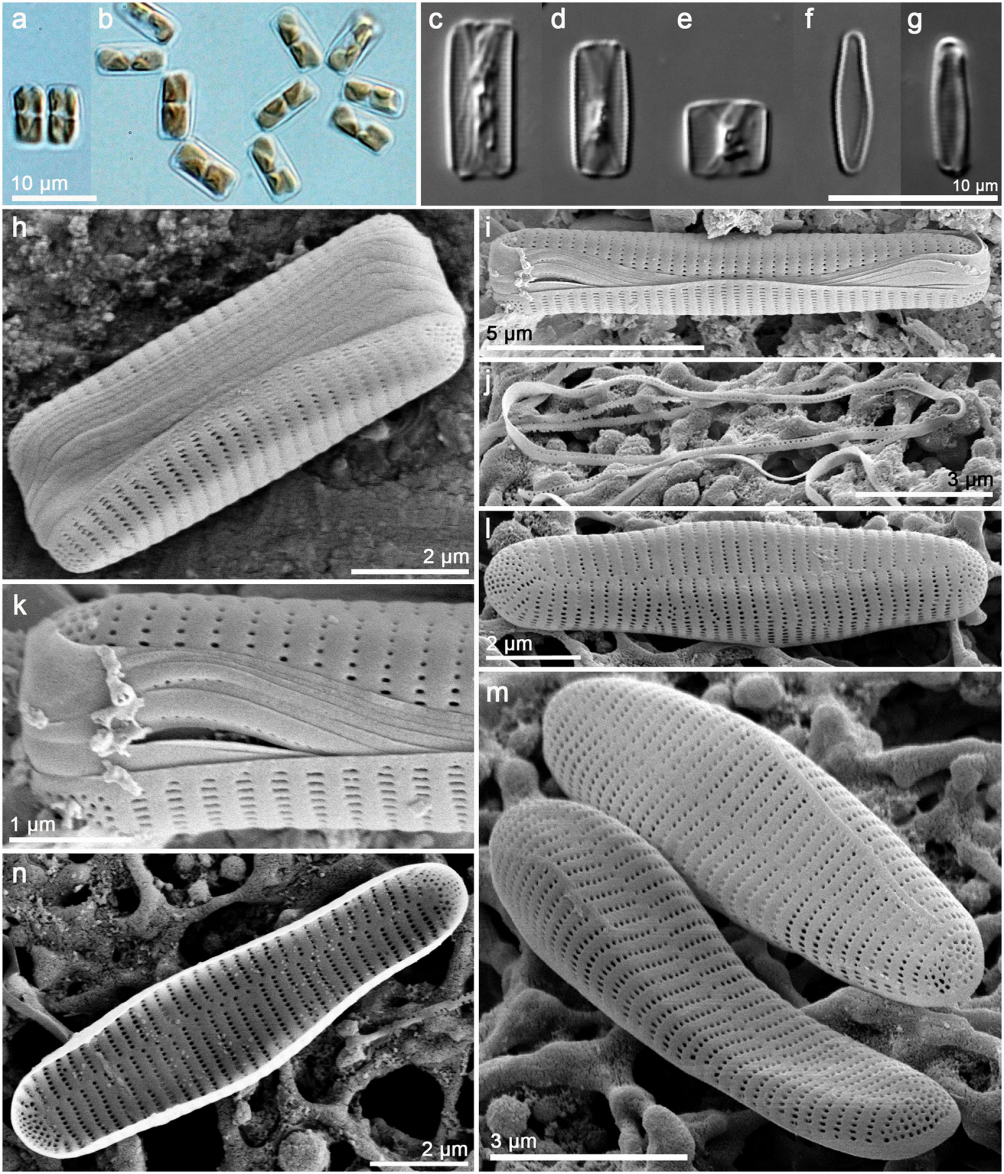
*Orizaformis holarctica*, light microscopy (LM) (a-g) and SEM (h-n). Cultured material SZCZCH111 (a-h and i-n) and natural material SZCZ20822 (l-k). (a) Two cells linked by valve face, two plastids per cell. (b) Ribbon-like colony linked by valve corner forming zigzag filament. (c-e) Cleaned material in girdle view. (f-g) Cleaned material in valve view. (h) Cleaned frustule in culture composed of numerous girdle bands. (I) Cleaned frustule from wild population. (k) porous girdle bands of wild population. (l-m) Exterior valve, parallel striae along a distinct sternum. (n) Interior valve with well-developed apical pore fields, rimoportulae absent.

**Fig 4:**
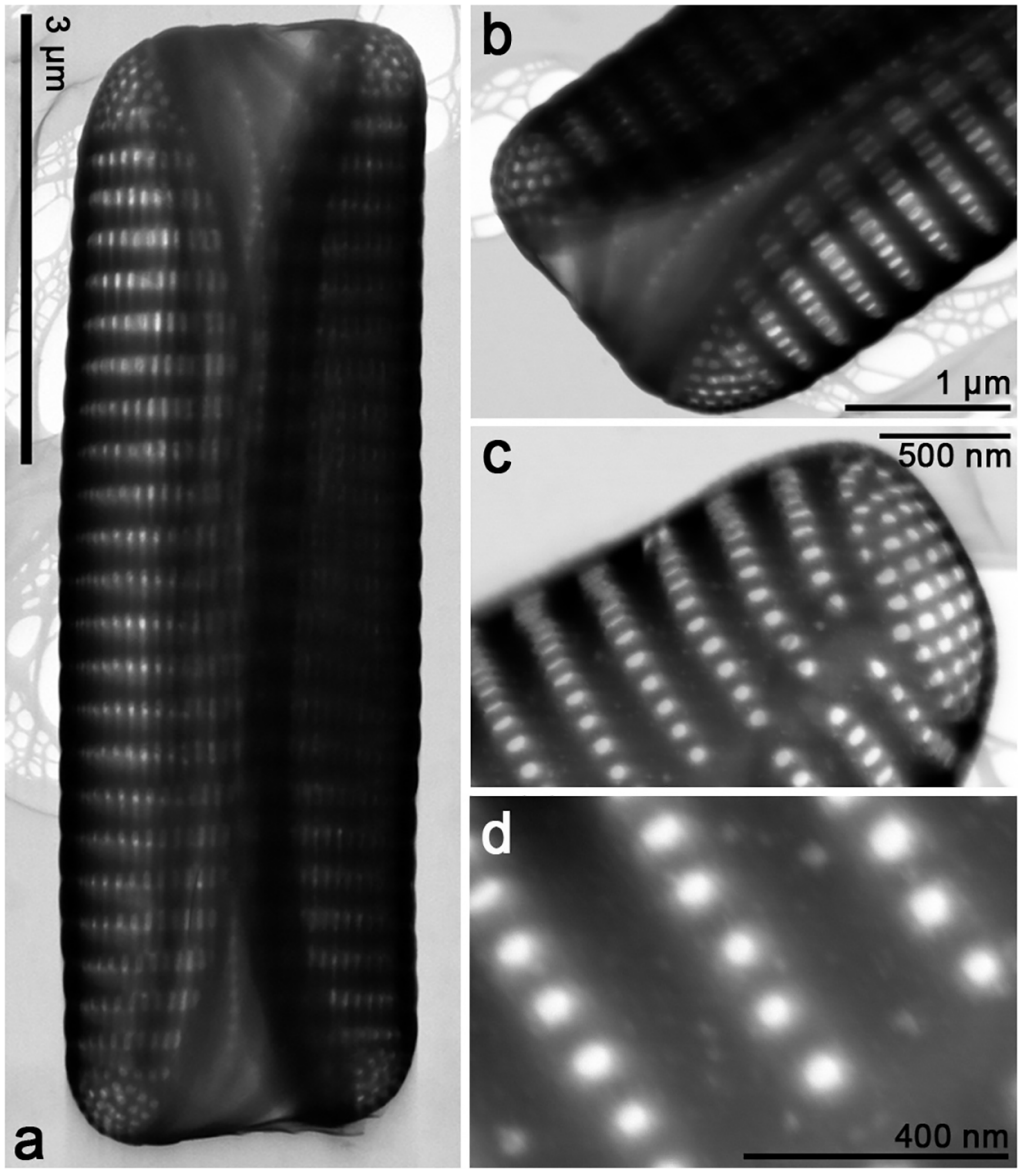
*Orizaformis holarctica*, TEM. Cultured material SZCZCH111. (a) Entire valve in girdle view. (b) Detail of the specimen illustrated in Fig 4a, it shows that girdle bands are composed of a single row of puncta with apically elongated areolae in the valve mantle. (c) Detail of the apex in the valve face. (d) Enlarged view of Fig 4c, showing circular areolae in the valve face.

*Orizaformis holarctica Witkowski*, *Chunlian Li & Ashworth sp*. *nov*. Figs 3 and 4:

Diagnosis: Valves linear to linear-elliptic with sometimes slightly expanded middle part and broadly rounded apices, 5.33–11.63 μm long, 2.25–2.39 μm broad, striae 37–40 in 10 μm in culture, 34 in 10 μm in natural population. Valve surface flat, sternum narrow and linear, transapical striae barely recognizable in LM.

Holotype: Based on cultured material SZCZCH111 deposited in Palaeoceanology Unit, Faculty of Geosciences, University of Szczecin and Figs 3 and 4 represent the holotype.

Isotype: slide BM 101 795 in the Natural History Museum, London, UK.

Paratype: culture CCMP143 (ex “*Bellerochea malleus*”) at the National Center for Marine Algae and Microbiota culture collection, USA and culture SZCZCH756 deposited in Palaeoceanology Unit, Faculty of Geosciences, University of Szczecin, Poland.

Type locality: the sediment of Wangfu Jiao Reef in the littoral zone of Changdao island near Yantai, China, Bohai Sea; 37°57.17' N 120°44.04' E. Collected by A. Witkowski, 26 June 2013.

Etymology: *holarctica* referring to the known distribution of the species in the marine part of the Holarctic plant realm.

Morphology: Frustules are rectangular in girdle view (Fig 3a–3e), and the girdle is composed of up to a dozen bands each with a single row of fine punctae (Figs 3i–3k, 4a and 4b). The valve external surface is flat, with an abrupt transition between valve face and the mantle. Mantle relatively deep (Fig 3h). Sternum linear, narrow (Fig 3i–3m). Transapical striae alternate and parallel throughout, forming shallow grooves that alternate with slightly elevated interstriae, 34–40 in 10 μm (Fig 3i–3m). Striae composed of small oblong areolae in the valve face and apically elongate areolae in the mantle (90–100 in 10 μm, Fig 4a–4d). Occlusions have not been observed thus far. Rimoportulae absent (Fig 3i and 3n). A relatively large pore field composed of apically oriented rows of puncta at both apices (Fig 4c).

The holotype of *Orizaformis holarctica* was isolated from the sediment of Changdao Island in Yantai (Shandong Province, Bohai Sea coast) and is maintained in the Szczecin culture collection (SZCZCH111). The second strain of the species has been maintained in the culture in Provasoli-Guillard National Center for Marine Algae and Microbiota at Bigelow Laboratory in United States as “*Bellerochea malleus”* CCMP 143. This clone was isolated from Muroran, Hokkaido, Japan (42°18' N, 140°58.8' E) in 1971. The third strain of the species was isolated from the rock of Moss Landing, CA, USA (36°48.6' N, 121°46.8' W) and is maintained in the Szczecin culture collection (SZCZCH756). This species is known only from the above localities.

Comparison with established taxa: Because of its very small size and valve structure, which is barely resolvable in LM, *Orizaformis holarctica* exhibits a gross similarity to a few araphid pennate genera. One of these is the plagiogrammacean genus *Psammoneis*, which forms ribbon-like colonies and has frustules that have a much broader girdle than *Orizaformis*. However, the striae in *Psammoneis* are coarser and resolvable in LM [1], whereas in *Orizaformis* they are barely resolvable in LM (34–40 in 10 μm). The girdle in *Orizaformis* is composed of punctate bands, whereas in *Psammoneis*, it is composed of solid, unperforated bands. *Orizaformis holarctica* can also potentially be confused with *Fragilaria amicorum* Witkowski & Lange-Bertalot but the latter has coarser striae and a narrower girdle than *Orizaformis*. *Orizaformis* and *F*. *amicorum* are distinct in EM; the latter species has elaborate areolae occlusions, whereas in *Orizaformis* occlusions are unknown. The first species has elongate, slit-like areolae, which in *Orizaformis* are circular in the valve face, becoming oblong in the mantle.

#### Revision of the genus Psammoneis

**Fig 5:**
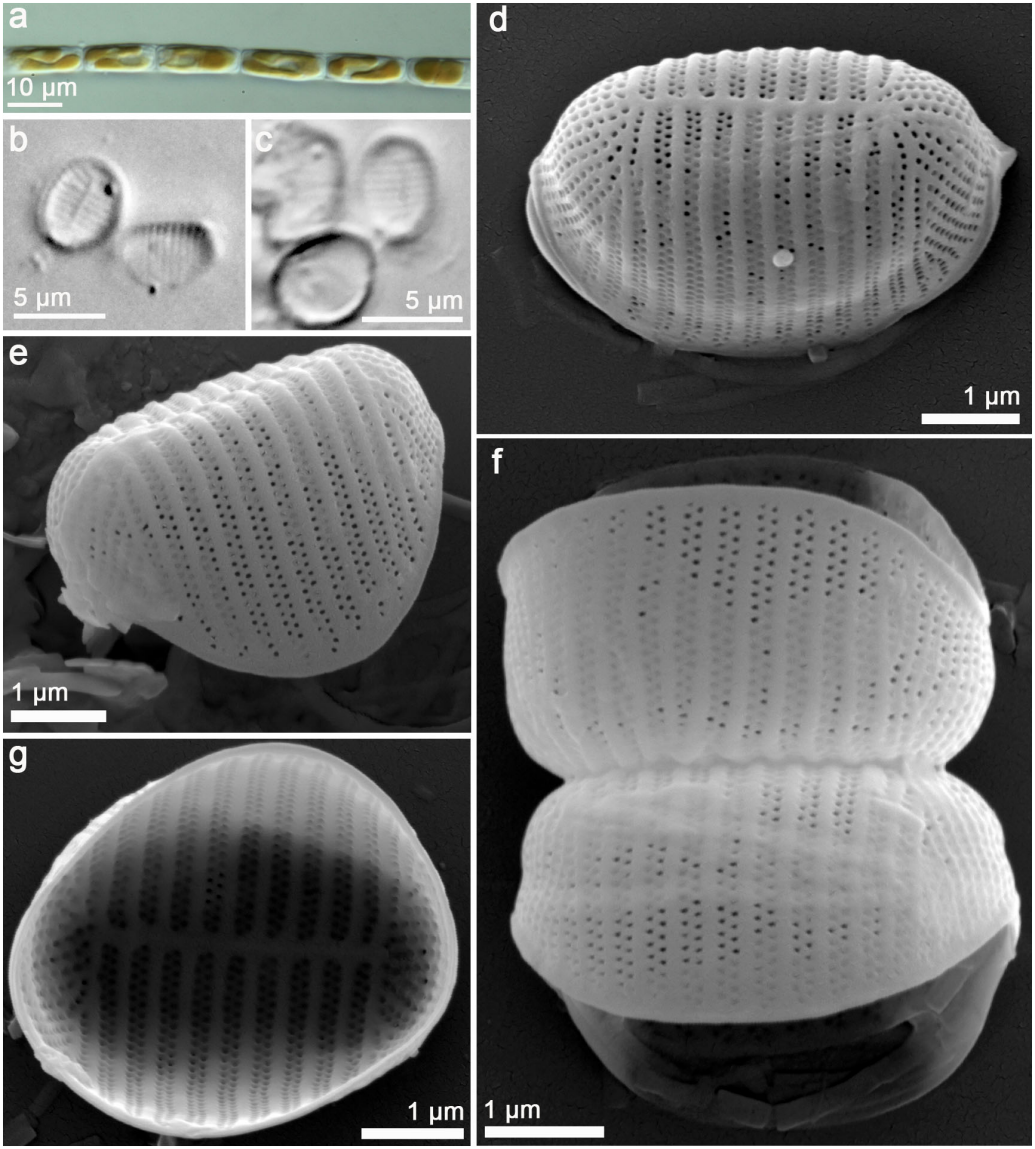
*Psammoneis obaidii*, LM (a-c) and SEM (d-g). Cultured material UTKSA0057. (a) Ribbon-like colony linked by valve face, two plastids per cell. (b-c) Cleaned material in valve view. (d) Exterior view, parallel striae arranged alternately across a distinct sternum and circular areolae in the valve face. (e) Tilted exterior view, showing circular areolae in the mantle. (f) Chain colony linked by valve face. (g) Internal view, rimoportula absent.

*Psammoneis obaidii Ashworth & J*. *Sabir sp*. *nov*. Fig 5:

Diagnosis: Cells elliptical to round in valve view, rectangular in girdle view. Length of valve is 3.5–6 μm, width 2.5–4 μm. Striae biseriate with alternating punctae, 8–11 per valve and becoming uniseriate and radiate around the apices to form the apical pore field. Valve mantle deep, with edge nearly straight or in a steep arc. Copulae without perforations. Two elongate plastids per cell lie on both the valve and girdle faces.

Holotype: UTKSA0057 “plagiogrammid A = *Psammoneis*” slide BM101790 in the Natural History Museum, London, UK. Cleaned material also available of this accession.

Type locality: Markaz Al Shoaibah (Al Qatan resort), Red Sea, Saudi Arabia (20°50.47' N, 39°24.05' E). Theriot Lab Red Sea Collection ID “SA12.” Collected by plankton net from shore by M. P. Ashworth, 19 January 2013.

Etymology: This species is named in honor of Professor Abdullah Obaid, Dean of the Faculty of Science at King Abdulaziz University, who was instrumental to our efforts in collecting, culturing and sequencing marine diatoms in Saudi Arabia.

Morphology: These small cells are often found in short colonies, joined valve face-to-valve face (Fig 5a and 5f). Plastids appear to be elongate, with two per cell (Fig 5a). Valves are elliptical in shape, but become almost round as the cell size decreases (Fig 5b and 5c). The sternum and interstice are broad and hyaline, lacking any sort of ornamentation. The apical pore fields are arranged in parallel rows of small circular porelli, spanning the apices of the valve face and extending beyond the margin and down the mantle to the valvocopula (Fig 5e). Striae are arranged in an alternating pattern across the sternum, and are biseriate with alternating pores (Fig 5d). There are no internal structures in the valve, including rimoportulae (Fig 5g). Girdle elements are thin and unperforated (Fig 5e).

Comparison with established taxa: In the light microscope, this taxon could be confused with *Psammoneis senegalensis* Sato, Kooistra & Medlin, because both taxa have small cells with a relatively thick sternum and interstriae alternating with striae across the sternum. The striae pores, which are hardly visible in the LM, are the key character in distinguishing these taxa; *P*. *senegalensis* possesses the linear pores typical in the rest of the genus, whereas *P*. *obaidii* has dual rows of alternating, circular pores. *Orizaformis* (described above) also possesses circular pores, they are uniseriate on the valve face, and grade into linear pores on the valve mantle. *Orizaformis* also has girdle bands with pores, which all *Psammoneis* spp. lack.

#### Revision of the genus *Neofragilaria*

*Neofragilaria* Desikachary, Prasad & Prema: Giffen (1980) described two species in the genus *Opephora* Petit from Mahé from the Seychelles Islands [12]. His taxonomic decision was based on LM examination of the microscopic slides and the fact that *Opephora* apparently was the closest in terms of morphology to his novel species. Based on analysis of the original material examined by Giffen and comparison with *Neofragilaria nicobarica* Desikachary, Prasad & Prema suggested that one of M. Giffen species and *N*. *nicobarica* may be conspecific. Indeed the size ranges overlap and the gross morphology of the two taxa in question is very similar. *Opephora anomala* as measured by M. Giffen is 25–28 μm long and 7–9 μm wide and has 5–6 transapical striae in 10 μm (Giffen 1980, p. 151), whereas Desikachary et al. (1987, p. 8) indicated that *N*. *nicobarica is* 15–35 μm long and of 8–10 μm wide with 6–7 striae in 10 μm [13]. Based on this information we propose a formal transfer of *Opephora anomala* into *Neofragilaria*. In terms of gross morphology apparently *O*. *lineata* Giffen also belongs in *Neofragilaria*. Its formal transfer is described below.

**Fig 6:**
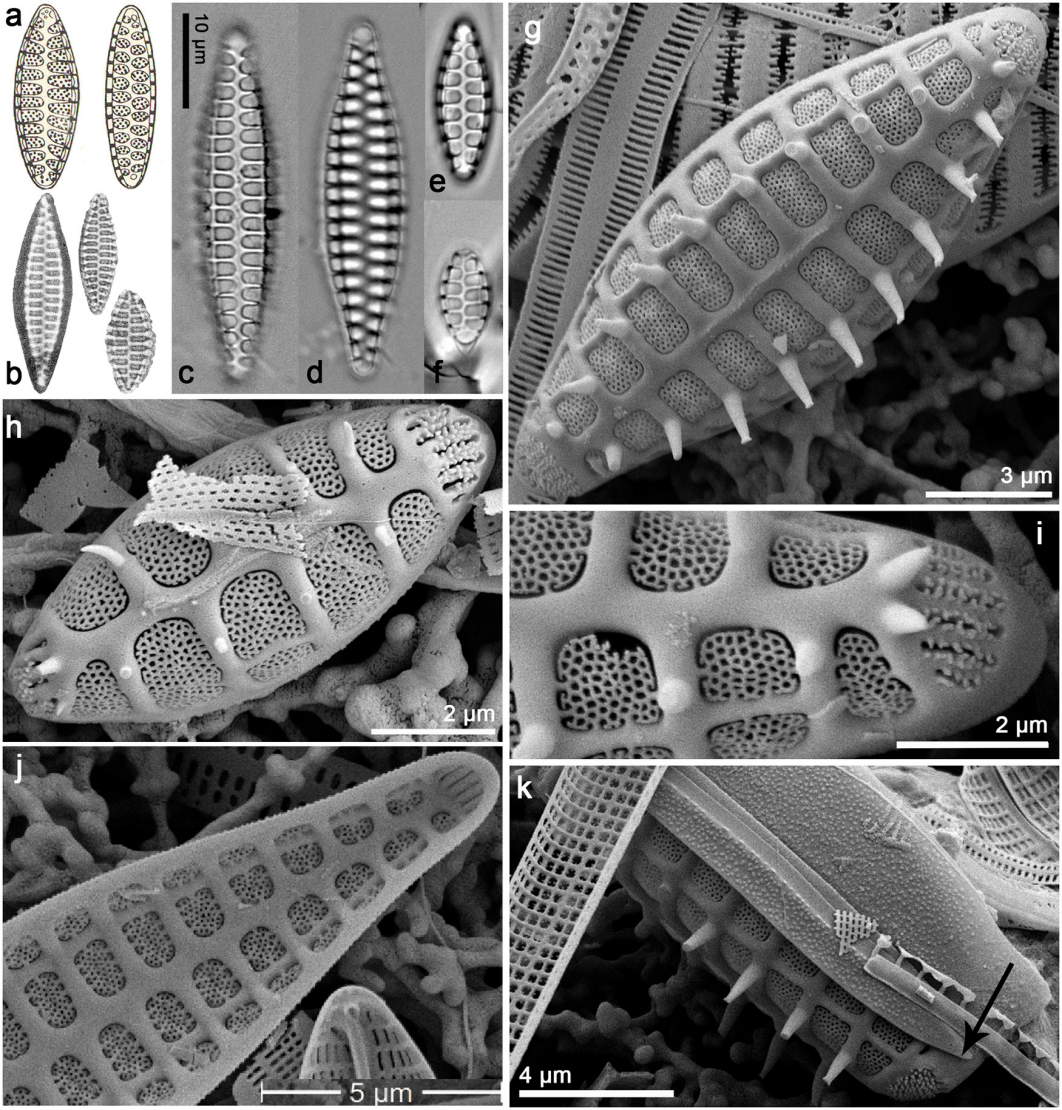
*Neofragilaria anomala*, drawings (Fig a), LM (b-f) and SEM (g-k). (a) Valve view, drawings of Malcolm Giffen, without scale. (b) Scan of illustrations from Desikachary et al (1987), showing robust striae and a distinct sternum. (c-f) Valve view, showing robust striae and a distinct sternum. These images were taken from Slide Br 426/52 of Malcolm Giffen depsoited in Hustedt Diatom Collection at Alfred Wegener Institute in Bremerhaven. (g-i) External view, based on clean material SZCZ16502 of natural samples from Juan de Nova in Mozambique Channel deposited in Palaeoceanology Unit, Faculty of Geosciences, University of Szczecin. (j) Internal view, the material is the same as Fig 6g–6i. (k) Incomplete girdle band (see arrow), the material is the same as Fig 6g–6i.

*Neofragilaria anomala (Giffen) Witkowski & Dąbek comb*. *nov*. Fig 6:

Basionym: *Opephora anomala* Giffen 1980. A checklist of marine littoral diatoms from Mahé, Seychelles Islands. Bacillaria 3, p. 150, fig. 33–35.

Synonym: *Neofragilaria nicobarica* Desikachary, Prasad & Prema 1987: 8, pl. 306: figs 9, 10. 12, 14

Our measurements of *Neofragilaria anomala* show that its size range is significantly larger than presented by either Giffen (1980) or Desikachary et al. (1987). Here we amend the size range of *N*. *anomala* to 8.0–35 μm long and 4–10 μm wide with 5–10 striae in 10 μm (Fig 6a–6k).

**Fig 7:**
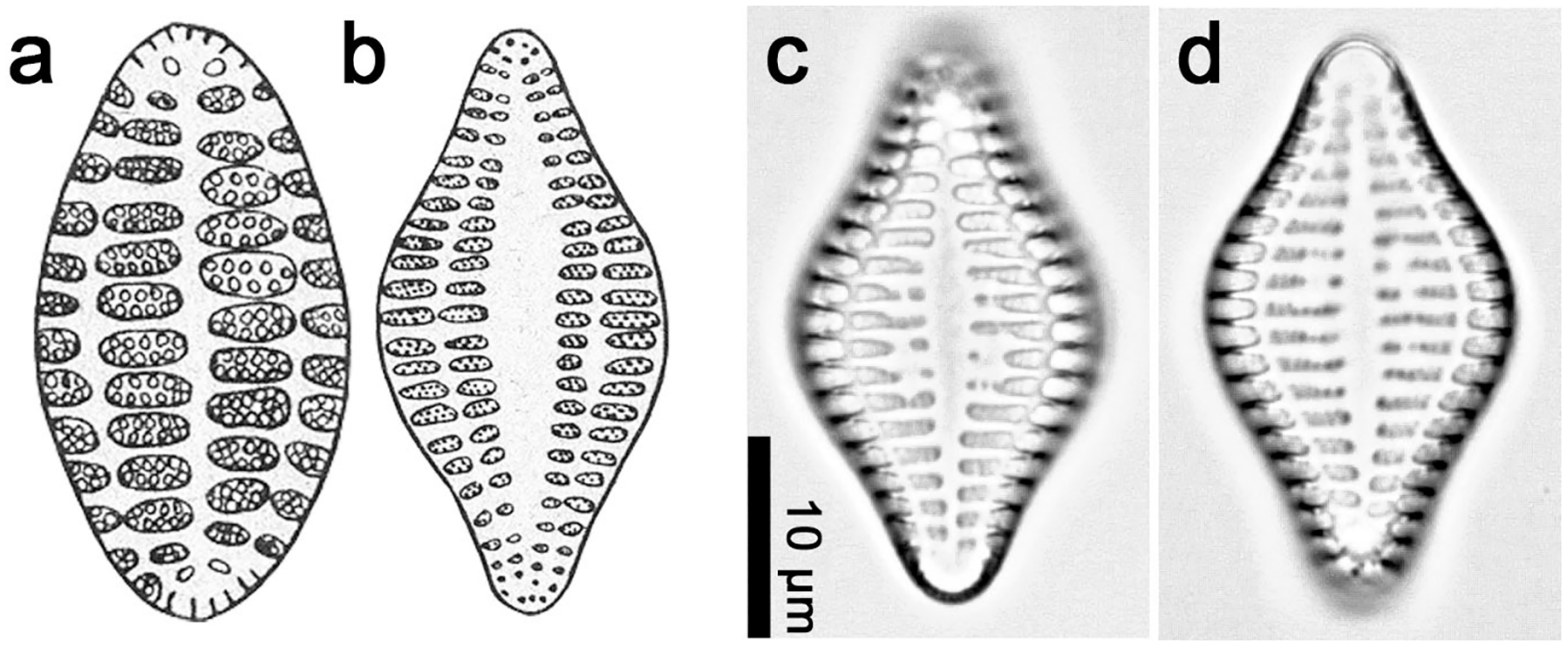
*Neofragilaria lineata*. (a-b) The valve view of drawings by Malcolm Giffen, without scale. (c-d) Valve view in LM, these images were taken from slide Br 426/53 deposited in Hustedt Diatom Collection at Alfred Wegener Institute in Bremerhaven.

*Neofragilaria lineata (Giffen) Witkowski & Dąbek comb*. *nov*. Fig 7:

Basionym: *Opephora lineata* Giffen 1980. A checklist of marine littoral diatoms from Mahé, Seychelles Islands. Bacillaria 3, p. 151, fig. 36, 37.

*Neofragilaria lineata* has rhombic-lanceolate valves with broadly rounded apices (Fig 7a–7d), 25–28 μm long, 14 μm wide. Sternum narrow linear to lanceolate(Fig 7a–7d). Transapical striae coarse (Fig 7a–7d), 8 in 10 μm. Whereas *N*. *anomala* is fairly widespread and known from warm waters across the oceans [3,13–14], *O*. *lineata* has only been reported thus far from the type habitat.

**Fig 8:**
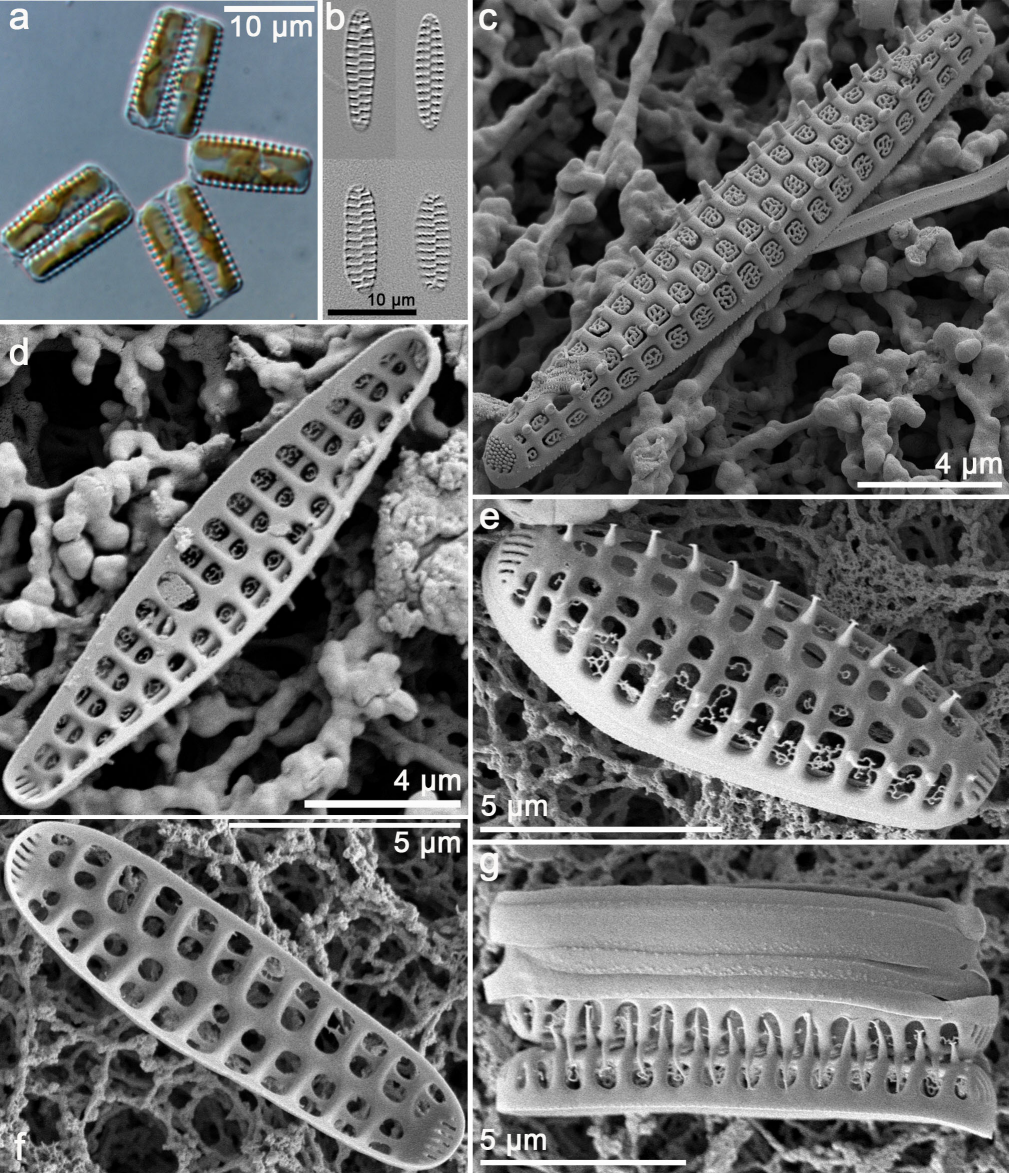
*Neofragilaria stilus*, LM (a-b) and SEM (f-g). Cultured material SZCZM116. (a) Zigzag colony linked by valve corners, frustule rectangular in girdle view, two plastids per cell. (b) Cleaned material in valve view, linear to lanceolate, robust striae. (c) Exterior view of natural sample, showing elaborate cribra and marginal spines. (d) Interior view of natural sample, a robust sternum, rimoportulae absent. (e) Exterior view in culture, showing corroded cribra and slit-like areolae in the apical pore field. (f) Interior view, rimoportulae absent. (g) Chain colony in girdle view and plain girdle bands.

**Fig 9:**
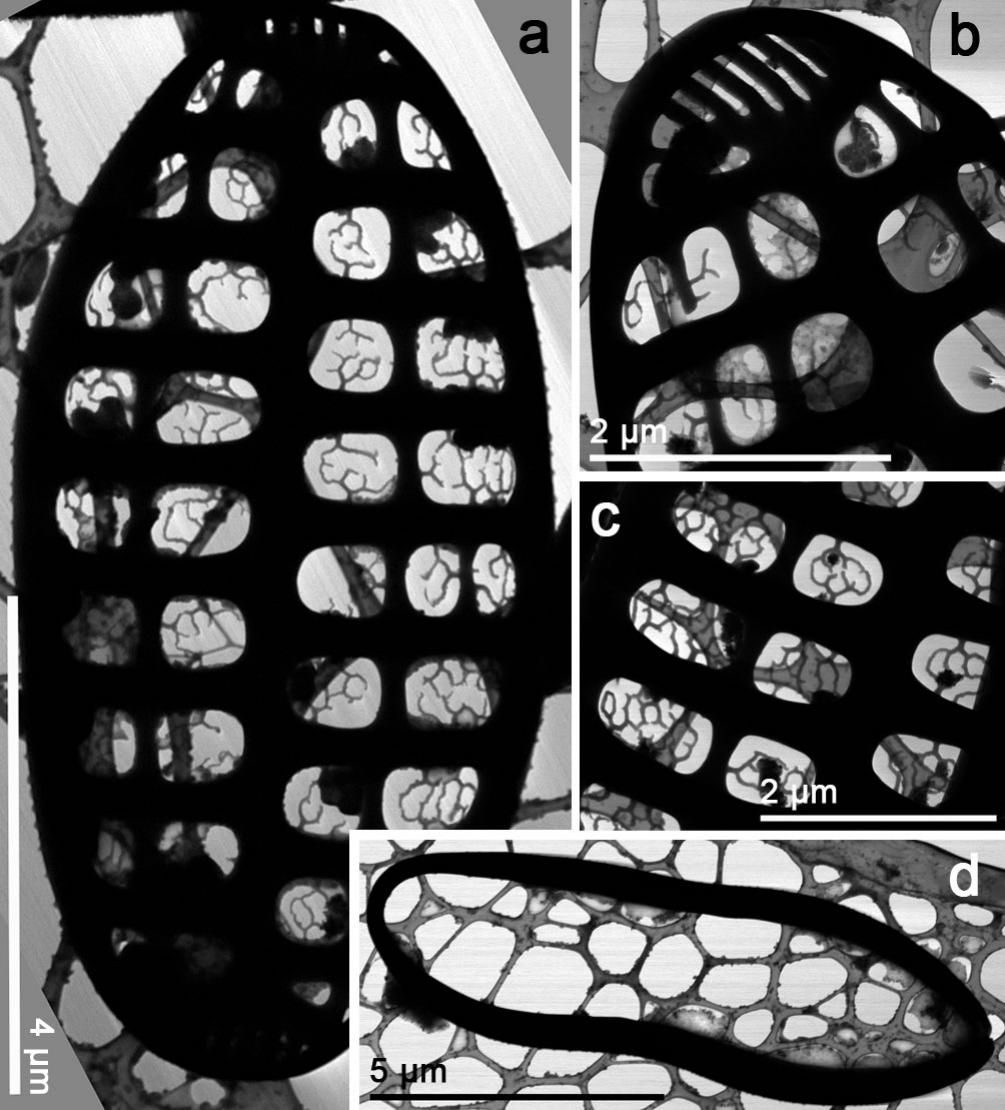
*Neofragilaria stilus*, TEM. Cultured material SZCZM116. (a) Entire valve in valve view. (b) Detail of the apex. (c) Enlarged view of Fig 9a. (d) A closed girdle band.

*Neofragilaria stilus Krzywda*, *Witkowski & Chunlian Li sp*. *nov*. Figs 8 and 9:

Diagnosis: Chloroplasts two per cell, running the full length of the valve face and turning slightly into the girdle region. Frustules rectangular in girdle view, forming zigzag colonies or united in aggregates. Valves in natural populations usually linear to lanceolate elongate with obtusely rounded apices, in culture rather linear elongate, sometimes opephoroid, with broadly rounded head pole and acutely rounded footpole, 6.5–13.3 (in culture), up to 18.9 (in natural population) μm long, 3–4.3 μm in width. Sternum distinct, with alternate striae. Striae coarse, parallel throughout, 10–12 in 10 μm composed of large areolae with a rotae hymene, attached with two pegs along the apical axis, two—three per single striae, visible as a single opening internally. T-shaped spines on the interstriae at the valve face-mantle junction.

Holotype: Cultured material SZCZM116 deposited in Palaeoceanology Unit, Faculty of Geosciences, University of Szczecin.

Isotype: slide BM 101 796 in the Natural History Museum, London, UK.

Type locality: from the coral sand from Bazaruto, 12 mile reef deep offshore bank, the coast of Mozambique, Indian Ocean; 21°21.3' S, 35°30.18' E. Collected by Dr. Rhett Bennett on March 9th 2013.

Etymology: *stilus* refers to the shape of the valve Latin—*stilus*–cigar (stick).

Morphology: Frustules are rectangular in girdle view with rounded apices (Fig 8a), and the girdle is composed of several solid, open and plain bands (Figs 8g and 9d). Frustules are united into ribbon-like colonies by means of long, bifurcate spines (Fig 8g). Valves in both natural population and in culture have flat to slightly arched valve faces, with relatively steep mantles (Fig 8c and 8e). The junction between the valve face and the mantle is delineated by an apically oriented bar (Fig 8c and 8e). The sternum is robust, with alternate striae, slightly elevated above the valve face (Fig 8c and 8e). The transapical striae are parallel throughout and composed of 2 to 3 large areolae (Fig 8b–8f). Each areola is occluded by a rota, which is connected to the interstriae by two simple pegs on the apical side of the areola (Figs 8c–8e and 9a–9c). The mantle areolae are larger than those on the valve face, and multiple pegs may anchor the rota (Fig 8c and 8d). One strongly bifurcated spine is positioned on each interstriae at the valve margin (Fig 8c and 8e). Pore fields at both apices are composed of up to 8 rows of single elongated slits with small outgrowths of silica along their external openings that appear peg like or like broken pores (Fig 8c–8f).

Comparison with established taxa:

*Neofragilaria stilus* resembles the smallest specimens of *N*. *anomala* (syn.: *N*. *nicobarica*). However, the two species can be differentiated by LM using size dimensions. *Neofragilaria stilus* is 6.5–18.9 μm long and 3–4.3 μm wide with 10–12 striae in 10 μm, whereas *N*. *anomala* is 8–35 μm long and 4–10 μm wide with 5–10 striae in 10 μm.

#### Revision of the genus *Talaroneis*

**Fig 10:**
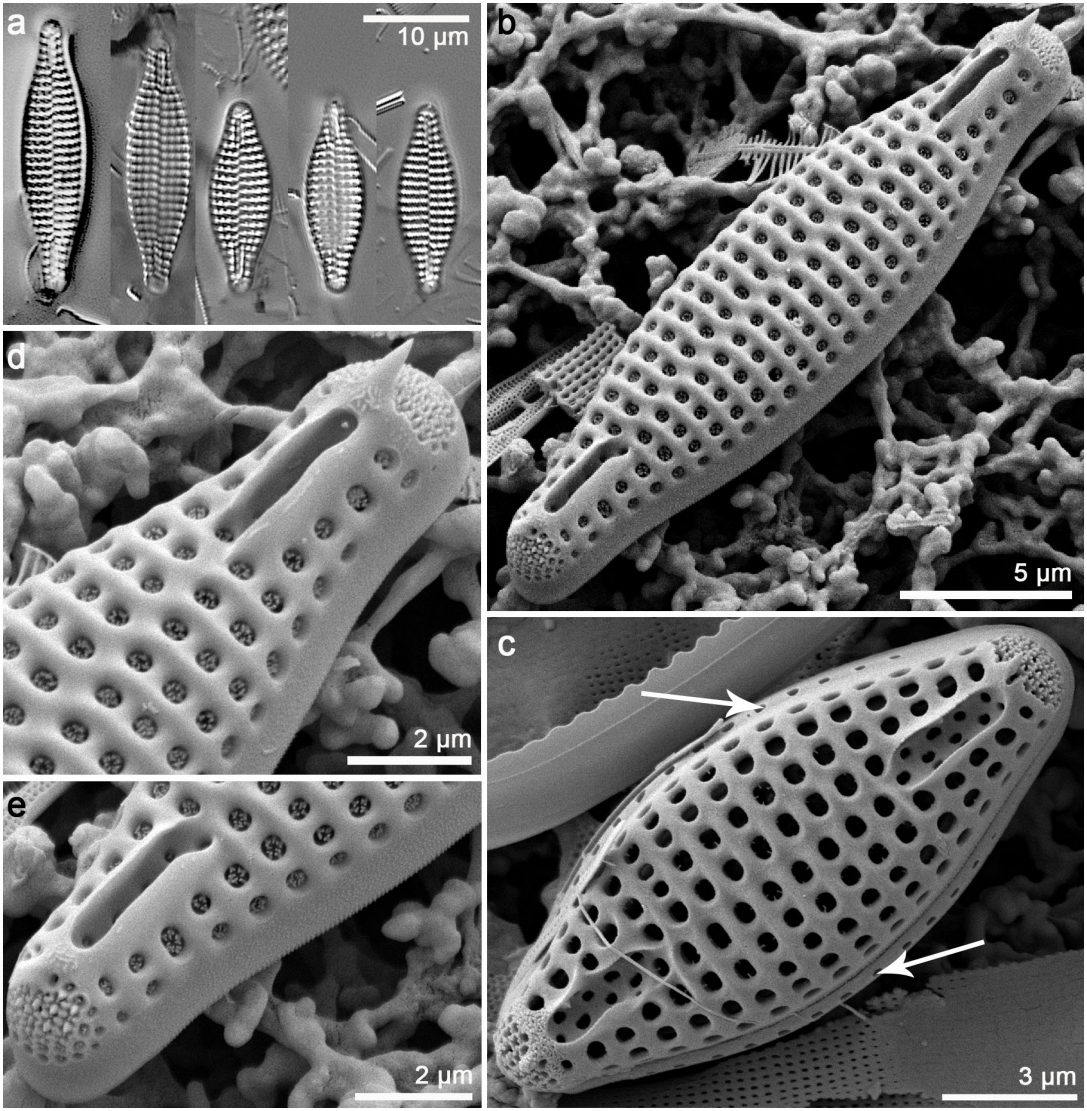
*Talaroneis biacutifrons*, LM (a) and SEM (b-e). Cleaned material SZCZ16205_1 from Juan de Nova in Mozambique Channel. (a) Cleaned material of valve view in different size. (b) Entire valve in valve view, showing subapical furrows on the apices and an acute spine located at each apex. (c) Entire valve view, valvocopula (see arrows) bearing a single row of puncta. (d) Detail of close ups of one apex of the specimen illustrated in Fig 10b. (e) Detail of the other apex of the specimen illustrated in Fig 10b

*Talaroneis biacutifrons Witkowski*, *Riaux-Gobin & Ruppel sp*. *nov*. Fig 10:

Synonym: *Dimeregramma fulvum sensu* Foged 1975, Fig. VIII: 5, 6, not *D*. *fulvum* sensu Prasad & Felgenhauer 1988, Figs 1–12.

**Fig 11:**
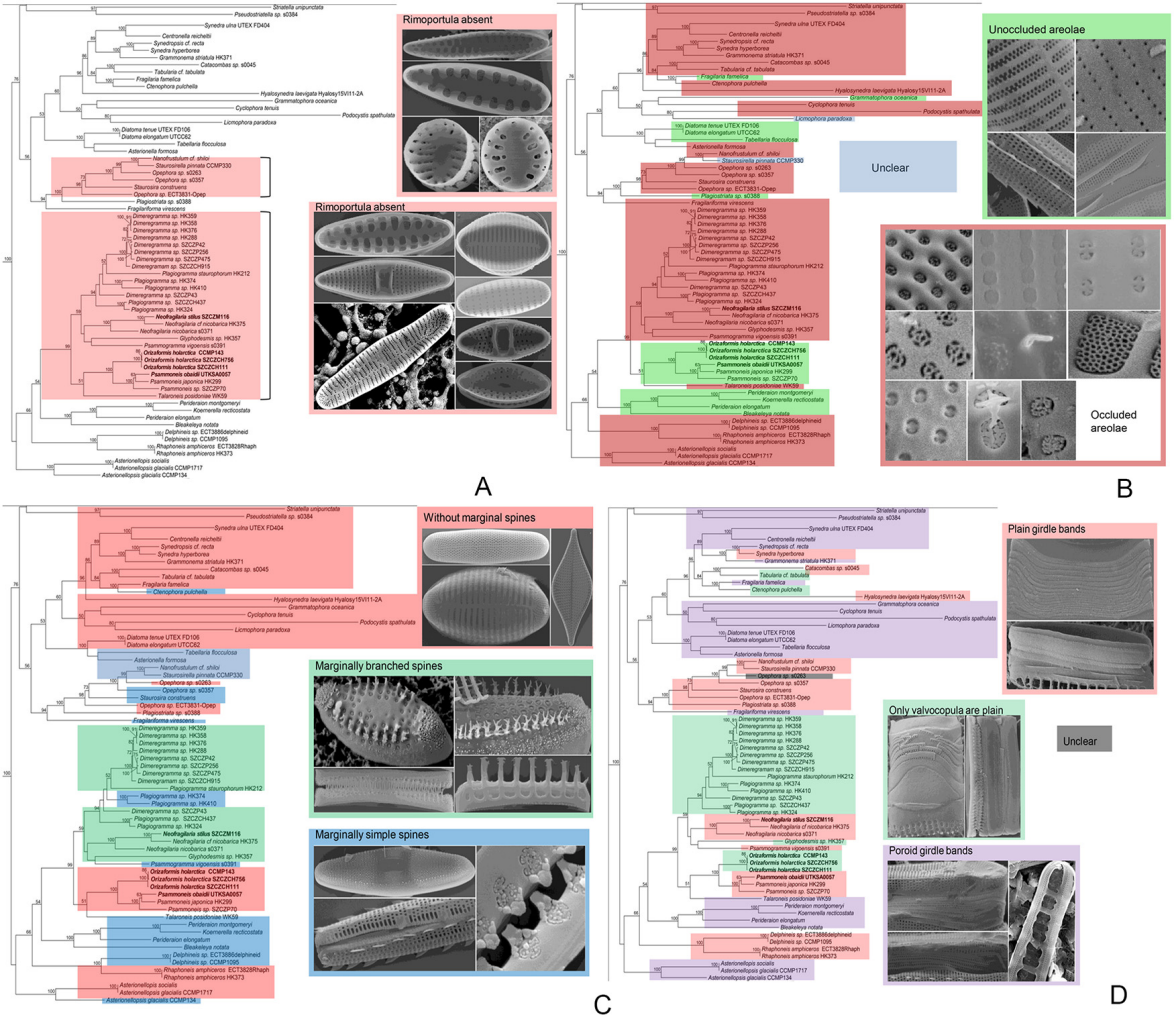
Mapping of morphological features in the araphid clade onto the three-gene tree. (A) Absence of rimoportulae. (B) Unoccluded or occluded areolae. (C) Morphology of marginal spines: without, simple, branched. (D) Girdle band morphology.

**Fig 12:**
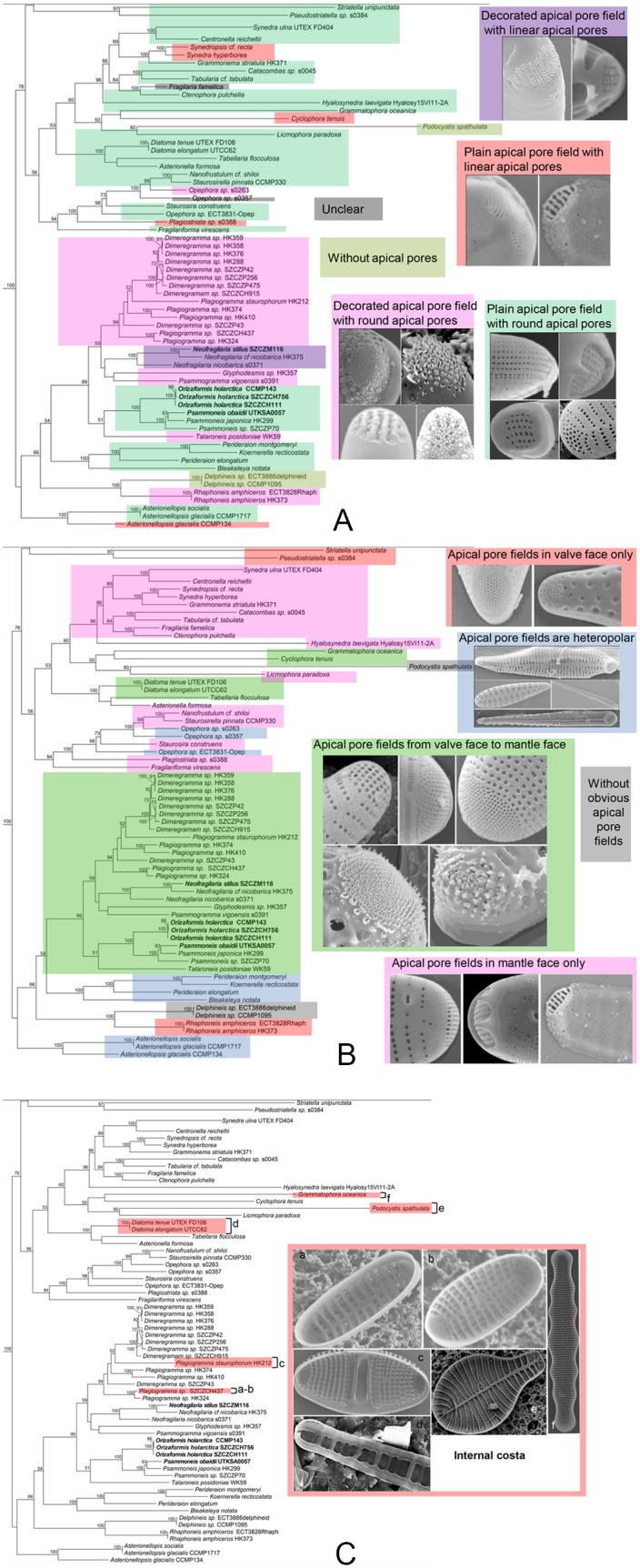
Mapping of morphological features in the araphid clade onto the three-gene tree. (A) The shape of apical pores and decorated or plain apical pore field. (B) Four kinds of apical pore fields: apical pore fields in the valve face only; apical pore fields in mantle face only; apical pore fields are heteropolar and apical pore fields from valve face to mantle face. (C) Internally thickened costae.

*Dimeregramma furcigerum sensu* Montgomery & Miller 1978, fig. 96G not *Talaroneis furcigerum* (Grun.) Sterr. = *Dimeregramma furcigerum* Grunow in Kooistra et al. Fig 1.

Diagnosis: Chloroplasts unknown. Valves linear elliptical to almost elliptical with slightly protracted (in longer specimens) to obtusely rounded (in short specimens) apices, 13–26 μm long, 5–7 μm wide. An acute spine at each apex. A shallow furrow, visible with LM, in the apical part of the valve sternum, which is perforated by areolae that are not aligned with the striae. Transapical, alternate striae coarse, parallel to slightly radiate at the apices, 12–13 in 10 μm. Areolae closed by multiple dendritic rota attached by small pegs to the areolae wall. Valvocopula bearing a single row of relatively large poroids (the entire cingulum not yet observed in SEM).

Holotype: SZCZ16205_1 from natural sample, deposited in Palaeoceanology Unit, Faculty of Geosciences, University of Szczecin. Isotype: slide BM BM 101 797 in the Natural History Museum, London, UK. Based on cleaned material from Juan de Nova in Mozambique Channel.

Type locality: in the coral sand from Juan de Nova atoll, a shoal 11–13.5 m deep, the Mozambique Channel, Indian Ocean; 16°59.34’ S, 42°47.37’ E. Collected by A. Witkowski on April 27th 2009.

Etymology: *biacutifrons* refers to the presence of the two acute spines on the apices.

Morphology: Valve face is flat, with a rather narrow sternum (Fig 10a). The valve face slopes gently towards the mantle. Transapical, alternate striae are parallel throughout and composed of relatively robust areolae (Fig 10b). Striae are formed by areolae occluded with dendritic rota (Fig 10b–10d and 10e). Complete girdle was not observed, although valvocopulae have been observed and show the presence of a single row of relatively large pores (Fig 10c) similar to *T*. *furcigerum* (Grunow) Sterrenburg. It differs from that species primarily by its shape (broadly ellipitical in ours vs. linear-lanceolate in theirs) but also by its striae density. It also differs from *D*. *fulvum sensu* Prasad & Felgenhauer, which based on our evaluation of their illustrations contains two different but clearly Dimeregramma spp. An acute spine occurs at each apex, positioned on the edge of the apical pore field (Fig 10b–10e). The apical pore field is composed of ca. 8 rows of apically oriented fine pores with some siliceous outgrowths around the external openings (Fig 10c).

Comparison with established taxa: *Talaroneis biacutifrons* could be mistaken for some of the small species closely related to *Grammonema striatulla* (Lyngbye) C. Agardh = *Fragilaria striatula* Lyngbye or *Fragilaria barbararum* Witkowski, Metzeltin & Lange-Bertalot but can be distinguished from those species by the clear indication of the apical groove in the sternum near the valve apex, which appears as a rather hyaline part of the valve with LM. *Talaroneis furcigerum* generally has longer valves (30–80 μm long, 7–9 μm wide) [7], with an indistinct sternum and with striae composed of small areolae, as opposed to the zig-zag sternum and larger areolae of *T*. *biacutifrons*. *Talaroneis posidoniae* is in general smaller than *T*. *biacutifrons* and has much finer striation (21–22 in 10 μm, versus 12–13 in 10 μm).

## Discussion

In this paper we describe a new genus, *Orizaformis* Witkowski, Chunlian Li. & Ashworth, and three new species, *Psammoneis obaidii* Ashworth & J. Sabir, *Neofragilaria stilus* Krzywda, Witkowski & Chunlian Li, *Talaroneis biacutifrons* Witkowski, Riaux-Gobin & Ruppel, and assign them all to the family Plagiogrammaceae on the basis of molecular data (except *Talaroneis biacutifrons*, which lacks molecular data) and the general morphological features shared by the Plagiogrammaceae [1]: an outline typical of pennate diatoms, possession of apical pore fields and a sternum, rimoportulae absent and areolae occluded by rotae, and add two new combination taxa, *Neofragilaria anomala* (Giffen) Witkowski & Dąbek, *Neofragilaria lineata* (Giffen) Witkowski & Dąbek.

*Orizaformis* differs from the rest of the genera in Plagiogrammaceae by its areolar structure with circular areolae not occluded by siliceous growths on the valve face changing to apically-elongate areolae on the mantle. This kind of shift in areolar structure has never been observed in the other genera in the family. Although *Orizaformis holarctica* is sister to *Psammoneis* in the phylogenetic tree with a high degree of support (bv = 100%) (Figs 1 and 2), the relatively long branch between *Orizaformis* and *Psammoneis* and the morphological differences are sufficient to recognize *Orizaformis holarctica* as a new genus, distinct from *Psammoneis*. *Psammoneis obaidi* groups with *Psammoneis japonica* with good support (Figs 1 and 2), however, it is distinct from the other described *Psammoneis* taxa in its biseriate striae and circular areolae. Relatively coarse transapical striae and costae in *Neofragilaria stilus* are typical characteristics within *Neofragilaria*. Phylogenies indicate that *Neofragilaria stilus* and *Neofragilaria nicobarica a*re sister taxa with high support (bv = 100%) (Figs 1 and 2), however, *N*. *stilis* can be separated from other taxa by its elaborate cribra, which are connected to the transapical costa by two simple outgrowths, whereas the areolae in *N*. *nicobarica* are occluded by a broad, plate-like cribra [3].

Most non-pennate and numerous araphid pennate diatoms possess rimoportulae [6], but rimoportulae have never been observed in Plagiogrammaceae. All araphid clade “A” taxa (Fig 1) possess a rimoportula except Plagiogrammacean diatoms (Fig 11A). As for the core araphids clade “B”, there are basically two major groups (Fig 1): a clade with rimoportulae including *Fragilaria* and *Synedra*, and a clade lacking rimoportulae, including *Nanofrustulum*, *Opephora*, *Staurosirella* and *Staurosira* (Fig 11A) [10,15–17]. However, other studies have shown taxa with rimoportulae mixed in this clade, such as *Fragilariforma* sp. in Medlin et al. [18] or *Plagiostriata goreensis* in Sato et al. [19]. In describing *Plagiostriata goreensis*, Sato et al. [19] noted that its rimoportulae were highly reduced, lacking the typically internal labiate-shaped morphology, and up to 10% of the valves lacked it entirely. The loss of rimoportulae as well as the position of *Plagiostriata goreensis* in the phylogenetic tree [19] suggested that *Plagiostriata goreensis* re-acquired its rimoportulae independently after the loss of the structure at the root of the clade. In our phylogeny (Fig 1) we recovered a sequential divergence of *Fragilariforma*, then *Plagiostriata*, suggesting a gradual loss of the rimoportulae. The complete structure of the rimoportulae in *Fragilariforma virescens* as compared to the reduced state in *Plagiostriata goreensis*is supports this conclusion. Furthermore, the plagiogrammacean clade and fragilariacean genera lacking rimoportulae do not occur in the same clade (Fig 11A), suggesting that the loss of rimoportulae in araphid diatoms occurred independently at least two times.

The material for mapping morphological features are from below clones: *Delphineis sp*. SZCZCH195, *Nanofrustulum sp*. SZCZCH194, *Neofragilaria stilus* SZCZM116, *Orizaformis holarctica* SZCZCH111, *Talaroneis biacutifrons* SZCZ16205_1, *Bleakeleya notata* HK247, *Glyphodesmis sp*. HK357, *Koernerella recticostata* HK242, *Nanofrustulum sp*. HK056, *Opephora sp*. HK296, *Plagiogramma sp*. HK212, *Plagiogramma sp*. HK324, *Plagiogramma sp*. HK374, *Perideraion montgomeryii* HK246, *Psammogramma vigoensis* s0391, *Psammoneis japonica* HK299, *Plagiogramma sp*. HK324, *Rhaphoneis amphiceros* HK373, *Grammatophora sp*. s0340, *Hyalosynedra sp*. s0359, *Opephora sp*. s0263, *Opephora sp*. s0308, *Opephroa sp*. s0357. *Diatoma tenuis* (cited from http://westerndiatoms.colorado.edu/taxa/species/diatoma_tenuis). All “SZCZ####” materials mentioned in this manuscript are deposited in Palaeoceanology Unit, Faculty of Geosciences, University of Szczecin, and are available upon request; All “HK####” materials mentioned in this paper are stored in the Theriot Lab collection at the University of Texas, Austin and are available by request from MPA; All s0### materials mentioned in this manuscript are stored in a collection at Fukui Prefectural University, Japan curated by S. sato, and are available upon request.

Several diagnostic characters have been proposed to define the Plagiogrammaceae but some of these have been questioned as more diversity within the family is uncovered. One such feature is the siliceous areolae occlusions. Most of the Plagiogrammaceae, as well as other araphid diatoms, possess elaborate areolae occlusions, but in two of the recently described genera—*Orizaformis* and *Psammoneis—*no occlusions have been observed thus far (Fig 11B). In the latter genus, Sato et al. [1] mentioned that a remnant of areolae occlusions has been detected in *P*. *pseudojaponica* but it still needs to be confirmed that these are remnants of siliceous areolae occlusions rather than organic residue or artifacts of SEM preparation. It is likely that there may have been a secondary loss within the family; areolae unoccluded by siliceous elements can also be found in many other lineages of centric diatoms and araphid pennate diatoms (Fig 11B).

Another character for defining the Plagiogrammaceae are marginal spines. In the Plagiogrammaceae clade, taxa in most genera, except *Psammoneis* and *Orizaformis* (Fig 11C), possess marginal spines, some of which can be highly branched. However, taxa with marginal spines are scattered throughout the araphid clades, so it is unlikely to be a synapomorphy for the Plagiogrammaceae.

Regarding girdle structure, there are several types within the Plagiogrammaceae. *Dimeregramma*, *Plagiogramma*, and *Glyphodesmis* all possess girdle bands bearing a single row of puncta, except for the hyaline valvocopula, which is noticeably larger than the other girdle elements (Fig 11D). Girdle elements of *Orizaformis* also have single rows of puncta and a hyaline valvocopula, though in this genus there is no noticeable size difference between the valvocopula and other girdle elements. In *Talaroneis*, all girdle elements have a single row of puncta, whereas in *Neofragilaria*, *Psammoneis* and *Psammogramma*, the girdle elements are unperforated.

*Dimeregramma*, *Plagiogramma*, *Glyphodesmis*, *Psammogramma* and *Talaroneis* possess decorated apical pore fields with round apical pores (Fig 12A), whereas *Orizaformis* and *Psammoneis* have round apical pores but with plain apical pore fields. These features are found in other araphid pennates. However, decorated apical pore fields with linear apical pores have only been observed in *Neofragilaria* (Fig 12A), which likely represents an apomorphy for this genus. Plain apical pore fields with linear apical pores have never been detected in Plagiogrammaceae.

The material for mapping morphological features are from below clones: *Dimeregramma sp*. SZCZP475, *Dimeregramma sp*. SZCZCH915, *Neofragilaria stilus* SZCZM116, *Orizaformis holarctica* SZCZCH111, *Opephora sp*. SZCZP696, *Plagiogramma sp*. SZCZCH437, *Podocystis sp*. SZCZ19909, *Asterionellopsis socialis* HK319, *Asterionellopsis socialis* HK181, *Cyclophora tenuis* HK216, *Grammatophora undutala* HK367, *Grammonema striatula* HK371, *Hyalosira sp*. ECT3907, *Hyalosynedra sp*. HK363, *Hyalosynedra laevigata* HK362, *Koernerella recticostata* HK242, *Neofragilaria sp*. HK375, *Perideraion montgomeryii* HK246, *Psammoneis sp*. HK299, *Plagiogramma sp*. HK324, *Plagiogramma staurophorum* HK212, *Synedropsis hyperborea* HK117, *Dimeregramma minor* s0355, *Neofragilaria nicobarica* s0372, *Opephora sp*. s0361, *Rhaphoneis sp*. s0263.

Although defining characteristics of Plagiogrammaceae have been proposed in previous studies [1,3,6–7], no synapomorphy for the family has been suggested. The most distinctive character of the Plagiogrammaceae constitutes the elaborate apical pore fields. However, as discussed previously, many diatoms have apical pore fields, including most araphid diatoms [20] and some raphid diatoms [6,21,22]. The apical pore fields of Plagiogrammaceae are large and well developed, extending from the valve face to the mantle (Fig 12B). This differs from the apical pore field structure found in other araphid diatoms, such as *Staurosira construens*, *Staurosirella pinnata*, *Fragilaria* spp. and *Synedra* spp. (Fig 12B), which are typically restricted to the valve margin or valve mantle only [6,23–25], or the apical pore fields of some other araphids that are located only in the valve face, as in *Striatella unipunctata* or *Rhaphoneis amphiceros*.

None of the character states associated to apical pore field morphology constitutes a synapomorphy for the Plagiogrammaceae. Pore fields that span the valve face and mantle are also found in unrelated araphid pennate clades including *Grammatophora oceanica*, *Cyclophora tenuis*, *Diatoma tenue* and *Tabellaria flocculosa*. As for the porelli “decorated” with spines and rims, these are also found in *Rhaphoneis amphiceros*, which may or may not be sister to the Plagiogrammaceae. These “decorations” have not been observed in *Psammoneis* or *Orizaformis*.

While we have not identified a morphological synapomorphy for the Plagiogrammaceae, the molecular data suggests the family is monophyletic (Figs 1 and 2). There does seem to be a morphological “gestalt” to the Plagiogrammacean genera—a distinct sternum, well-developed apical pore fields that span the valve face to the valve mantle and a lack of a rimoportula, which helps to distinguish them from other small araphid genera such as *Staurosira*, *Pseudostaurosira*, *Opephora* and *Delphineis*. At this point, these similarities should be viewed as strictly phenetic and further study may reveal a synapomorphy for the family, perhaps from developmental or ultrastructural data.

Williams et al. [26] suggested that a natural classification of diatoms should be based on the concept of monophyly. Our unconstrained three-gene analysis (Fig 1) recovers *Dimeregramma* and *Plagiogramma* each as not monophyletic because of an unresolved trichotomy of *Dimeregramma* (SZCZP43) with a *Plagiogramma* clade (HK374, HK410). Similarly the four-gene analysis recovers *Dimeregramma* embedded within *Plagiogramma* (Fig 2). However the SH test demonstrates that neither unconstrained trees (Figs 1 and 2) are significantly different from the constraint trees (S1 and S2 Figs) in which *Plagiogramma* and *Dimeregramma* are reciprocally monophyletic. Therefore we cannot confidently reject monophyly for either on the basis of our molecular data and taxon sampling.

Based on observed morphology, previous descriptions of *Dimeregramma* and *Plagiogramma* are likewise ambiguous. In describing *Plagiogramma*, Greville (1859) [27] characterized the genus as “frustules quadrangular, two or more united into a filament; valves linear or elliptical; striae moniliform, vittae two or more, pervious, parallel with the striae”. Greville’s “vittae” refer to the transverse costae, which he later points out form a “stauros” found in all species in this genus. This central transverse hyaline area (which we will refer to as a “fascia” rather than the “stauros” of raphid pennate diatoms) and transverse costa [3,9] have become the main diagnostic character of *Plagiogramma*, despite the fact that those structures are not unique to this genus. There are other pennate diatoms that exhibit a similar fascia, e.g., the araphid diatom *Cyclophora tenuis* Castracane [3,28], the genus *Hustedtiella* Simonsen [3,29], and even selected species in some raphid pennate genera/species, e.g. *Planothidium lanceolatum* [30] and species of *Gliwiczia* Kulikovskiy, Lange-Bertalot & Witkowski [31], implying that this structure has evolved independently several times. In terms of internal costae, they also are present in other araphid diatoms (Fig 12C). Therefore, although these costae represent a useful character to distinguish *Plagiogramma* from *Dimeregramma* and other small, rimoportule-less taxa, we should not view this feature as synapomorphic.

As for *Dimeregramma*, it was characterized by the following description: “frustules quadrangular, two or more together; valves scarcely broader than front view, having the transverse costae or striae interrupted by a smooth, longitudinal median line [32].” Again, the costae are specifically mentioned here, but SEM observations (S3 Fig) show just how different these structures are from those of *Plagiogramma*. Rather than internal walls that completely cross the valve, the “costae” of *Dimeregramma* are external thickenings of the valve wall between the striae (“interstriae”). These thickened interstriae do not extend across the sternum. They are also where the marginal spines are found. The flattened, plate-like morphology of the marginal spines found near the apical pore fields occur in many *Dimeregramma* species, as well as in some of *Plagiogramma* species. It should be noted that *Talaroneis* species can also have thickened interstriae and elongate “flaps” of silica near the apical pore fields (Fig 10). The position of these taxa in different “subclades” of the Plagiogrammaceae (Figs 1 and 2), however, suggests these are not shared traits among these genera. As for the “smooth, longitudinal median line” on the sternum, this structure varies widely across *Dimeregramma* taxa (S3 Fig) and a wide, conspicuous sternum is present in many unrelated araphid pennate genera (S4 Fig).

Although none of these characters can be viewed as true synapomorphies for *Plagiogramma* and *Dimeregramma*, they are useful for distinguishing these closely related genera from each other and they help to distinguish them from other small araphid diatoms with apical pore fields and without rimoportula. Importantly, we also have not identified any characters which would unequivocally argue against reciprocal monophyly. Thus we elect to retain the present classification at this time, recognizing that neither genus has strong support for monophyly.

Based on our morphological studies, we propose the following key for the genera of Plagiogrammaceae:

1a Hyaline area on valve face, either transverse or apically oriented fascia………………………………………………….2
1b Sternum or a trace of sternum present with no other fascia on valve……………………………………………………3
2a Hyaline rim around part of valve-face apical pore field, raised above valve surface………………………………………………………………*Glyphodesmis*
2b Uninterrupted apical pore field without any rim……………………………4
3a Distinct areolae visible on valve face in LM…………………………………5
3b Individual areolae on valve face not visible in LM…………………………6
4a Central hyaline area present across apical axis, without internal tranverse costae…………………………………………*Dimeregramma*
4b Internal tranverse costae and central fascia present……………………………………………*Plagiogramma*
5a Pores in apical pore field round, two silica flaps parallel to valve margin proximal to apical pore fields……………………………………………*Talaroneis*
5b Pores in apical pore field linear……………………………………*Neofragilaria*
6a Areolae in striae round and occluded………………………*Psammogramma*
6b Areolae in striae appear unoccluded……………………………………7
7a Pores in striae linear or biseriate over entire valve…………….*Psammoneis*
7b Pores in striae round on valve face, grading to linear on margin……………………………………………….*Orizaformis*

Clade A and Clade B are now formally described as new subclasses in the Class Bacillariophyceae [33].

## Materials and Methods

### Sample collection and cultures

Living cells of *Orizaformis holarctica* sp. nov. were collected from the sediment of Wangfu Jiao Reef, on June 26th 2013 in the littoral of Changdao island (37°57.17' N 120°44.04' E), the south coast of Bohai Sea in Yantai region, NE China. No specific permission was required for this location and sampling activities, because this location is a public place and there is not endangered and protected species. *Neofragilaria stilus* sp. nov. was collected from the Indian Ocean deep off shore near 12 Miles Reef, Bazaruto, Mozambique on March 9th 2013. The status of this sampling site is not protected and it does not require any specific sampling permissions. *Psammoneis obaidii* was collected using a 20 μm mesh plankton net from the coast of the Red Sea in Saudi Arabia (20°50.47' N, 39°24.05' E) on January 19th 2013. Regarding this location, we had the collaboration with King Abdulaziz University KAU, which is the only governmental University in Jeddah that has got access to the Red Sea, so we normally do not need a permission to collect samples. *Talaroneis biacutifrons* was collected from Juan de Nova atoll in Mozambique Channel (16°59.34’ S, 42°47.37’ E) the Western Indian Ocean on April 27th 2009. The (Terres Australes et Antarctiques Françaises) (TAAF) gave the permission to conduct the study on this sampling site. Single cells were isolated from wild samples to obtain monoclonal cultures. *Orizaformis holarctica* and *Neofragilaria stilus* were grown in enriched seawater in 35 PSU f/2medium [34] at 18°C under a 16h:8h light:dark cycle, illuminated with 50 μmol m^−2^ s^−1^ of white light. *Psammoneis obaidii* was grown in enriched seawater in 40 PSU f/2 medium, at 27°C under a 12h:12h light:dark cycle, illuminated with 21 μE m^−2^ s^−1^ of white light.

### Microscopic examination

To observe the chloroplast morphology of live cells, clones were photographed in counting chambers using a Nikon TS300 inverted microscope (Nikon Corporation, Tokyo, Japan) equipped with a x100 Plan Apochromat oil immersion objective (n.a. = 1.40) equipped with differential interference contrast (DIC) optics. For morphological observations of cleaned valves in the light (LM) and electron microscopes (SEM and TEM), cell suspensions of cultures were boiled with 30% hydrogen peroxide at 150°C for a few hours. For wild samples, 10% hydrochloric acid was also added to remove calcium carbonate. Later the samples were washed with deionized water five times and boiled at 250°C for a few hours in 30% hydrogen peroxide to remove organic matter. Boiled cultures and wild samples were rinsed with deionized water five times. Eventually, cleaned diatom material was pipetted onto coverslips, dried and mounted on glass slides using Naphrax® (Brunel Microscopes Ltd, Wiltshire, U.K.). LM observations were made with a Zeiss Axio Imager M2 (Carl Zeiss, Jena, Germany) using a x100 PlanApochromatic oil immersion objective (n.a. = 1.46) equipped with DIC. For SEM examination, a few drops of cleaned material were put onto Whatman Nuclepore polycarbonate membrane filters (Fisher Scientific, Schwerte, Germany). When the membranes were dried, they were mounted onto aluminum stubs and coated with gold-palladium or gold for ca. 3–5 minutes. SEM and TEM observations were done at the Warsaw University of Technology, Faculty of Materials Science and Engineering using a Hitachi SEM/STEM S-5500 and SU8000. Additional SEM observations were carried out by means of Hitachi S-4500 at Goethe University in Frankfurt am Main, Germany. SEM observations of *Psammoneis obaidii* and *Orizaformis holarctica* strain CCMP143 were carried out at University of Texas in Austin under the same SEM protocol outlined in Ashworth et al. [10, 16].

### DNA extraction and PCR

DNA extraction and amplification protocols for *Psammoneis obaidii* at the University of Texas, Austin follow Ashworth et al. [16]. For the remainder of the species, DNA was extracted at the University of Szczecin. Depending on cell density, several milliliters of cell suspension from an exponentially growing culture were centrifuged for 15 min at 8,000 rpm to extract the genomic DNA using the Genomic DNA NucleoSpin®Plant II Kit (Macherey-Nagel, Germany) according to the manufacturer`s instructions. The small subunit (SSU) of ribosomal RNA, and two chloroplast genes (*rbc*L ad *psb*C) were amplified using the primers and the protocols described in Theriot et al. [35]. PCR amplification of the D1/D2 regions of the nuclear 28S rDNA was done using the primers and protocols as described in Scholin et al. [36]. PCR products were visualized in 1% agarose (Maximus, Poland) gel and then purified using Exonuclease I & Polar-BAP (EURx, Gdańsk, Poland) protocol. PCR products were sent to oligo.pl DNA Sequencing Laboratory IBB PAS, Warsaw, Poland for Sanger sequencing with use of BigDye Terminator v. 3.1 chemistry and ABI3730 xl sequencer.

### Phylogenetic analyses

Maximum likelihood (ML) analysis was performed with a three-gene (SSU, *rbc*L and *psb*C) dataset (S1 Dataset) using two stains of *Bolidomonas pacifica* (L. Guillou & M.-J. Chrétiennot-Dinet) as the outgroup. Because of the historical uncertainty as to whether or not the Plagiogrammaceae were pennates or not, we included several radial and polar centrics to test the general placement of the Plagiogrammaceae and the general instability of sister relationships to pennate diatoms. Another dataset (S2 Dataset), mainly focusing on Plagiogrammaceae with four markers (SSU, LSU, *rbc*L and *psb*C) using *Asterionella formosa* as outgroup, was also analyzed. The second structural alignment of SSU primary sequences was aligned by SSU-align using covariance models. The ambiguous sites with a PP less than the default of 0.9 were removed. The second structural alignment of LSU was aligned in the ARB program using maximum primary and secondary structural similarity in the ARB program (Technical University of Munich, Germany). GenBank accession numbers of the taxa used in this paper are listed in S1 Table. In both analyses, all of the datasets were partitioned by different genes, different codon positions (in case of chloroplast markers), and paired and unpaired sites (in case of SSU markers) with a GTR+G+I model. Phylogenetic trees were conducted with 1000 bootstrap replicates using rapid Bootstrap analysis in RAxML v8.1 [37]. The best-scoring ML trees were chosen as the final trees and bootstrap values were added to the nodes. For the constrained analyses, *Dimeregramma* and *Plagiogramma* in both datasets were constrained into separate, monophyletic groups. ML trees were estimated using the same procedure as for the unconstrained trees. The Shimodaira-Hasegawa (SH) test [38] was performed to compare the best constrained trees and the best unconstrained trees with RAxML v8.1.

### Nomenclature Acts

The electronic version of this article in Portable Document Format (PDF) in a work with an ISSN or ISBN will represent a published work according to the International Code of Nomenclature for algae, fungi, and plants, and hence the new names contained in the electronic publication of a PLOS ONE article are effectively published under that Code from the electronic edition alone, so there is no longer any need to provide printed copies. The online version of this work is archived and available from the following digital repositories: PubMed Central, LOCKSS.

## Supporting Information

S1 Table Strain collection information and GenBank accession numbers of the diatom taxa used in the phylogenetic analyses in this manuscript. Newly generated sequences are listed in bold.
(DOC)

S1 Fig Constrained maximum likelihood tree inferred from three genes. Maximum likelihood phylogeny of 161 diatoms (with bootstrap values at nodes) inferred from a concatenated alignment of SSU, *rbc*L and *psb*C markers, which constrained *Dimeregramma* and *Plagiogramma* both as a separate, monophyletic clade. The bold taxa are newly-described species. Support values lower than 50% were not included in the tree. Two *Bolidomonas pacifica* strains were used as outgroups.
(TIF)

S2 Fig Constrained maximum likelihood tree inferred from four genes. Constrained maximum likelihood tree consisting of 26 strains of Plagiogrammacean diatoms (with bootstrap values at nodes) inferred from four markers (LSU and SSU rDNA, *rbc*L and *psb*C), forcing *Dimeregramma* and *Plagiogramma* into monophyly. The bold taxa are newly-described species. Support values lower than 50% were not included in the tree. The araphid diatom *Asterionella formosa* was used as the outgroup.
(TIF)

S3 Fig The morphology variability of the sternum in *Dimeregramma* taxa. (a-b) *Dimeregramma sp*. (material of Malcom Giffen, slide No. 628). (c) *Dimeregramma sp*. HK358. (d) *Dimeregramma sp*. HK376. (e) *Dimeregramma sp*. SZCZP475. (f) *Dimeregramma sp*. HK288. (g) *Dimeregramma sp*. SZCZCH915.
(TIF)

S4 Fig The morphology variability of the sternum in *Staurosira*-like taxa. (a) *Staurosira sp*., natural material of SZCZ7386. (b) *Punctastriata sp*., natural material of SZCZ504. (c) *Punctastriata sp*., natural material of SZCZ503. (d) *Pseudostaurosira sp*., natural material of SZCZ7113. (e) *Staurosira venter*, natural material of SZCZ7113. (f) *Staurosira construens*, natural material of SZCZ5007. (g) *Staurosira sp*., natural material of SZCZ7113. (h) *Pseudostaurosira sp*., natural material of SZCZ7113.
(TIF)

S1 Dataset Data for Fig 1. Alignment of concatenated DNA sequence data (SSU rDNA, *rbc*L and *psb*C) in NEXUS format used for three-gene phylogenetic analysis in this study.
(ZIP)

S2 Dataset Data for Fig 2. Alignment of concatenated DNA sequence data (SSU, LSU, *rbc*L and *psb*C) in NEXUS format used for four-gene phylogenetic analysis in this study.
(ZIP)
