# Supplementary material for: New Insights into Plagiogrammaceae (Bacillariophyta) Based on Multigene Phylogenies and Morphological Characteristics with the Description of a New Genus and Three New Species
Source: PLoS One. 2015 Oct 14;10(10):e0139300. doi: 10.1371/journal.pone.0139300 (PMC4605507; doi:10.1371/journal.pone.0139300)
Supplement: S1 Table — Newly generated sequences are listed in bold. (DOC) [file pone.0139300.s001.doc]

| **Species** | **Strain** | **GenBank Accession** | | | |
| --- | --- | --- | --- | --- | --- |
|  |  | **SSU** | ***rbc*L** | ***psb*C** | **LSU** |
| [*Achnanthes coarctata*](http://www.ncbi.nlm.nih.gov/nuccore/HQ912594.1) Brébisson ex W. Smith | UTEX FD185 | HQ912594 | HQ912458 | HQ912287 | **-** |
| [*Achnanthes* sp.](http://www.ncbi.nlm.nih.gov/nuccore/KC309617.1) | SanNicAchnan | KC309473 | KC309545 | KC309617 | **-** |
| [*Achnanthes* sp.](http://www.ncbi.nlm.nih.gov/nuccore/KC309617.1) | ECT3684 | KC309476 | KC309548 | KC309620 | **-** |
| [*Achnanthes* sp.](http://www.ncbi.nlm.nih.gov/nuccore/KC309617.1) | ECT3911 | KC309475 | KC309547 | KC309619 | **-** |
| *Asterionella formosa* Hassall | [UTCC605](http://www.ncbi.nlm.nih.gov/nuccore/HQ912633.1) | HQ912633 | HQ912497 | HQ912326 | **-** |
| *Asterionellopsis glacialis* (Castracane) Round in Round, R.M.Crawford & D.G.Mann | CCMP1717 | HQ912646 | HQ912510 | HQ912339 | **-** |
| *Asterionellopsis glacialis* (Castracane) Round in Round, R.M.Crawford & D.G.Mann | CCMP134 | HQ912613 | HQ912477 | HQ912306 | **-** |
| *Asterionellopsis socialis* (J.C.Lewin & R.E.Norris) R.M.Crawford & C.Gardner | [ECT3920Asterionell](http://www.ncbi.nlm.nih.gov/nuccore/JX413579.1) | JX413545 | JX413562 | JX413579 | **-** |
| [*Bacillaria paxillifer*](http://www.ncbi.nlm.nih.gov/nuccore/HQ912627.1) (O.F. Müller) T. Marsson | UTEX FD468 | HQ912627 | HQ912491 | HQ912320 | **-** |
| *Berkeleya rutilans* (Trentepohl ex Roth) Grunow | ECT3616 | HQ912637 | HQ912501 | HQ912330 | **-** |
| *Biddulphia tridens* (Ehrenberg) Ehrenberg | ECT3838 | HQ912674 | HQ912538 | HQ912365 | **-** |
| *Biddulphiopsis membranacea* (Cleve) von Stosch & Simonsen | [ECT3655-Biddulphiopsis](http://www.ncbi.nlm.nih.gov/nuccore/HQ912638.1) | HQ912638 | HQ912502 | HQ912331 | **-** |
| *Biddulphiopsis titiana* (Cleve) von Stosch & Simonsen | ECT3697 | HQ912641 | HQ912505 | HQ912334 | **-** |
| *Biremis panamae* Barka, Witkowski, Weisenb. | P136 | KM078661 | KM078662 | KM078668 | **-** |
| *Bleakeleya notata*(Grunow) Round | UT HK#247 | HM627330 | HM627327 | HM627324 | *-* |
| *Bolidomonas pacifica* L.Guillou & M.-J.Chrétiennot-Dinet | p380 | AB430618 | AB430698 | **-** | **-** |
| *Bolidomonas pacifica* L.Guillou & M.-J.Chrétiennot-Dinet | CCMP1866 | HQ912557 | HQ912421 | HQ912251 | **-** |
| *Caloneis lewisii* Patrick | UTEX FD54 | HQ912580 | HQ912444 | HQ912273 | **-** |
| *Campylodiscus clypeus* (Ehrenberg) Ehrenberg ex Kützing | L951 | HQ912412 | HQ912398 | HQ912384 | **-** |
| *Campylodiscus* sp. Ehrenberg ex Kützing | 3613.8 | HQ912413 | HQ912399 | HQ912385 | **-** |
| *Catacombas sp.* (Bory de Saint-Vincent) D.M.Williams & Round | s0045 | **KR048195** | **KR048217** | **KR048229** | **-** |
| *Centronella reicheltii* [**Voigt**](https://www.google.pl/url?sa=t&rct=j&q=&esrc=s&source=web&cd=1&cad=rja&uact=8&ved=0CCAQFjAA&url=http%3A%2F%2Fwww.algaebase.org%2Fspeciesdetail.lasso%3Fspecies_id%3D33272&ei=bEjbVJjwCZbhatGggtAE&usg=AFQjCNHYEAGZ8lsXg7-ApOnCaB25CcbESg&sig2=wSqOGW6hnyGOfYs2BSfjiA) | [CCAP1011/1](http://www.ncbi.nlm.nih.gov/nuccore/HQ912635.1) | HQ912635 | HQ912499 | HQ912328 | **-** |
| [*Climaconeis riddleae*](http://www.ncbi.nlm.nih.gov/nuccore/HQ912644.1) A.K.S.K.Prasad | ECT3724 | HQ912644 | HQ912508 | HQ912337 | **-** |
| *Cocconeis placentula* Ehrenberg | UTEX FD23 | HQ912592 | HQ912456 | HQ912285 | **-** |
| *Cocconeis* sp. | ECT3901 | KC309479 | KC309551 | KC309622 | **-** |
| *Cocconeis stauroneiformis* (W. Smith) Okuno | s0230 | AB430614 | AB430694 | **-** | **-** |
| *Coscinodiscus concinnus* W.Smith Ireland | ECT3839 | HQ912681 | HQ912545 | HQ912369 | **-** |
| *Coscinodiscus granii* Gough | ECT3836-Coscinodisc | HQ912667 | HQ912531 | HQ912359 | **-** |
| *Coscinodiscus radiatus* Ehrenberg | CCMP310 | HQ912560 | HQ912424 | HQ912254 | **-** |
| *Coscinodiscus wailesii* Gran & Angst | ECT3847-Coscinodi | HQ912668 | HQ912532 | HQ912360 | **-** |
| *Craticula cuspidata* (Kutzing)D.G.Mann | UTEX FD35 | HQ912581 | HQ912445 | HQ912274 | **-** |
| *Ctenophora pulchella* (Ralfs ex Kützing) D.M.Williams & Round | [UTEX FD150](http://www.ncbi.nlm.nih.gov/nuccore/HQ912611.1) | HQ912611 | HQ912475 | HQ912304 | **-** |
| Cyclophora tenuis Castracane | ECT3723 | HQ912660 | HQ912524 | HQ912353 | **-** |
| *Cyclostephanos dubius* (Hustedt) Round | [Waco5](http://www.ncbi.nlm.nih.gov/nuccore/HQ912575.1) | HQ912575 | HQ912439 | HQ912268 | **-** |
| *Cyclotella meneghiniana* Kützing | Waco1 | HQ912576 | HQ912440 | HQ912269 | **-** |
| *Cyclotella sp.* (Kützing) Brébisson | [LO4-2](http://www.ncbi.nlm.nih.gov/nuccore/HQ912625.1) | HQ912625 | HQ912489 | HQ912318 | **-** |
| *Cylindrotheca closterium* (Ehrenberg) Reimann & J. Lewin | CCMP1855 | HQ912645 | HQ912509 | HQ912338 | **-** |
| *Cymatopleura elliptica* (Brébisson) | L1333 | HQ912659 | HQ912523 | HQ912352 | **-** |
| *Cymatosira belgica* Grunow | ECT3892Cybel | **-** | KC309563 | KC309635 | **-** |
| *Cymatosira lorenziana Grunow* | ECT3874cymatosiroi | KC309490 | KC309562 | KC309634 | **-** |
| *Delphineis sp. G.W.Andrews* | ECT3886delphineid | JX413544 | JX413561 | JX413578 | **-** |
| *Delphineis sp.* G.W.Andrews | CCMP1095 | HQ912629 | HQ912493 | HQ912322 | **-** |
| *Denticula kuetzingii* Grunow | UTEX FD135 | HQ912610 | HQ912474 | HQ912303 | **-** |
| *Diatoma elongatum* (Lyngbye) C.Agardh | UTCC62 | HQ912622 | HQ912486 | HQ912315 | **-** |
| *Diatoma tenue* C.Agardh | UTEX FD106 | HQ912593 | HQ912457 | HQ912286 | **-** |
| *Dimeregramma sp.* | HK358 | JX401231 | JX401249 | JX401267 | **KT192078** |
| *Dimeregramma sp.* | HK359 | JX401232 | JX401250 | JX401268 | **KT192079** |
| *Dimeregramma sp.* | HK376 | KF701596 | KF701605 | KF701614 | **KT192081** |
| *Dimeregramma sp.* | HK288 | JN975244 | JN975258 | JN975273 | **KT192075** |
| *Dimeregramma sp.* | SZCZP256 | **KR048190** | **KR048210** | **KT119340** | **KR048201** |
| *Dimeregramma sp.* | SZCZP42 | **KR048187** | **KR048208** | **KR048223** | **KT119336** |
| *Dimeregramma sp.* | SZCZP475 | **KR048189** | **KR048207** | **KR048222** | **-** |
| *Dimeregramma sp.* | SZCZP43 | **KT119334** | **KR048209** | **KT119342** | **KR048200** |
| *Dimeregramma sp.* | SZCZCH915 | **KT119332** | **KT119337** | **-** | **-** |
| [*Diploneis subovalis*](http://www.ncbi.nlm.nih.gov/nuccore/HQ912597.1)Cleve | UTEX FD282 | HQ912597 | HQ912461 | HQ912290 | **-** |
| *Entomoneis ornata* (Ehrenberg) Ehrenberg | 14A | HQ912411 | HQ912397 | HQ912383 | **-** |
| *Entomoneis* sp. | CS782 | HQ912631 | HQ912495 | HQ912324 | **-** |
| *Epithemia argus* (Ehrenberg) Kützing | CH211 | HQ912408 | HQ912394 | HQ912380 | **-** |
| *Epithemia sorex* Kützing | CH148 | HQ912409 | HQ912395 | HQ912381 | **-** |
| *Epithemia turgida* (Ehrenberg) Kützing | CH154 | HQ912410 | HQ912396 | HQ912382 | **-** |
| *Eunotia bilunaris* (Ehrenberg) Schaarschmidt | UTEX FD412 | HQ912599 | HQ912463 | HQ912292 | **-** |
| *Eunotia glacialis* Meister | UTEX FD46 | HQ912586 | HQ912450 | HQ912279 | **-** |
| *Eunotia pectinalis* (Kützing) Rabenhorst | NIES461 | HQ912636 | HQ912500 | HQ912329 | **-** |
| *Eunotia* sp. Ehrenberg | ECT3676 | KC309480 | KC309552 | KC309623 | **-** |
| *Fallacia monoculata* (Hustedt) D.G.Mann | UTEX FD254 | HQ912596 | HQ912460 | HQ912289 | **-** |
| *Fallacia pygmaea* (Kützing) | UTEX FD294 | HQ912605 | HQ912469 | HQ912298 | **-** |
| *Fistulifera saprophila* (Lange-Bertalot & Bonik) Lange-Bertalot | TCC508 | KC736618 | KC736593 | **-** | **-** |
| *Fragilaria famelica* [**(Kützing) Lange**](https://www.google.pl/url?sa=t&rct=j&q=&esrc=s&source=web&cd=1&cad=rja&uact=8&ved=0CCAQFjAA&url=http%3A%2F%2Fwww.algaebase.org%2Fsearch%2Fspecies%2Fdetail%2F%3Fspecies_id%3D56333&ei=DEnbVOfdLtfbap6ygoAH&usg=AFQjCNFi3t2BcFYkfzi75Wi1fnKAOCwdxQ&sig2=XU-k0YbzyjB8YlRVXEcXjg) | [UTEX FD255](http://www.ncbi.nlm.nih.gov/nuccore/HQ912588.1) | HQ912588 | HQ912452 | HQ912281 | **-** |
| *Fragilariforma virescens* (Ralfs) D.M.Williams & Round | [UTEX FD291](http://www.ncbi.nlm.nih.gov/nuccore/HQ912628.1) | HQ912628 | HQ912492 | HQ912321 | **-** |
| *Glyphodesmis sp.* williamsonii (W.Smith) Grunow | HK357 | **-** | JX401248 | JX401266 | **-** |
| *Gomphonema affine* Kützing | UTEX FD173 | HQ912608 | HQ912472 | HQ912301 | **-** |
| *Gomphonema parvulum*(Kützing) Kützing | UTEX FD241 | HQ912595 | HQ912459 | HQ912288 | **-** |
| *Grammatophora oceanica* Ehrenberg | [CCMP410](http://www.ncbi.nlm.nih.gov/nuccore/HQ912634.1) | HQ912634 | HQ912498 | HQ912327 | **-** |
| *Grammonema striatula* | HK371 | KF701591 | KF701600 | KF701609 | **-** |
| *Gyrosigma acuminatum* (Kützing) Rabenhorst | UTEX FD317 | HQ912598 | HQ912462 | HQ912291 | **-** |
| [*Halamphora coffeaeformis*](http://www.ncbi.nlm.nih.gov/nuccore/KJ463509.1) (C.Agardh) Levkov | 7977-AMPH101 | KJ463449 | KJ463479 | KJ463509 | **-** |
| [*Hantzschia amphioxys* var. *major*](http://www.ncbi.nlm.nih.gov/nuccore/HQ912404.1) Grunow | A4 | HQ912404 | HQ912390 | HQ912376 | **-** |
| *Hyalosynedra laevigata* (Grunow) D.M.Williams & Round | Hyalosy15VI11-2A | JX401235 | JX401253 | JX401271 | **-** |
| *Koernerella recticostata* (Körner) M.P.Ashworth, C.S.Lobban & E.C.Theriot | UT HK#242 | HM627331 | HM627328 | HM627325 | **-** |
| *Lemnicola hungarica*(Grunow) F.E. Round & P.W. Basson | UTEX FD456 | HQ912626 | HQ912490 | HQ912319 | **-** |
| *Licmophora paradoxa* (Lyngbye) C.Agardh | [CCMP2313](http://www.ncbi.nlm.nih.gov/nuccore/HQ912612.1) | HQ912612 | HQ912476 | HQ912305 | **-** |
| *Lithodesmioides polymorpha* Stosch | [ECT3772-Lithodesmioides](http://www.ncbi.nlm.nih.gov/nuccore/HQ912655.1) | HQ912655 | HQ912519 | HQ912348 | **-** |
| *Lithodesmium intricatum* Ehrenberg | [ECT3850](http://www.ncbi.nlm.nih.gov/nuccore/HQ912678.1) | HQ912678 | HQ912542 | HQ912368 | **-** |
| *Mastodiscus radiatus* (J.W.Bailey) A.K.S.Prasad | ECT3822 | HQ912675 | HQ912539 | HQ912366 | **-** |
| *Mayamaea permitis* (Hustedt) K. Bruder | TCC540 | KC736630 | KC736600 | **-** | **-** |
| *Melosira nummuloides* C.Agardh | CCMP482 | HQ912566 | HQ912430 | HQ912260 | **-** |
| *Melosira sp.* [**varians C.Agardh**](https://www.google.pl/url?sa=t&rct=j&q=&esrc=s&source=web&cd=7&ved=0CDkQFjAG&url=http%3A%2F%2Fwww.algaebase.org%2Fspeciesdetail.lasso%3Fspecies_id%3D31650&ei=HWDbVLOwOsHOaPrpgogP&usg=AFQjCNELCyF-fCg7ApirjQ9BXvBNbCX-ew&sig2=dArLQq0W6CdG9zXO1O4v7Q&bvm=bv.85761416,d.bGQ) | TN-2014 BayCutMelosira | KJ577861 | KJ577898 | KJ 577932 | **-** |
| *Melosira varians* C.Agardh | ECT3833Melo | KC309539 | KC309611 | KC309682 | **-** |
| *Meuniera membranacea* (Cleve) P.C.Silva in Hasle & Syvertsen | ECT3896 | KC309482 | KC309554 | KC309624 | **-** |
| *Minutocellus polymorphus* (Hargraves & Guillard) Hasle, Stosch, & Syvertsen | ECT3920Minutoce | KC309498 | KC309572 | KC309645 | **-** |
| *Minutocellus polymorphus* (Hargraves & Guillard) Hasle, Stosch, & Syvertsen | CCMP497 | HQ912568 | HQ912432 | HQ912262 | **-** |
| *Nanofrustulum cf. shiloi* | [CCMP2649](http://www.ncbi.nlm.nih.gov/nuccore/HQ912578.1) | HQ912578 | HQ912442 | HQ912271 | **-** |
| *Navicula cari* Ehrenberg | AT-82.04 | AM501991 | AM710457 | **-** | **-** |
| *Navicula cryptocephala* Kützing | UTEX FD109 | HQ912603 | HQ912467 | HQ912296 | **-** |
| *Navicula reinhardtii* Grunow | AT-124.15 | AM501976 | AM710442 | **-** | **-** |
| *Navicula tripunctata (*O.F.Müller) Bory de Saint-Vincent | AT-202.01 | AM502028 | AM710495 | **-** | **-** |
| *Neidium affine* (Ehrenberg) Pfizer | UTEX FD127 | HQ912583 | HQ912447 | HQ912276 | **-** |
| *Neidium bisulcatum* (Lagerstedt) Cleve | UTEX FD417 | HQ912591 | HQ912455 | HQ912284 | **-** |
| *Neidium productum* (W. Smith) Cleve | UTEX FD116 | HQ912582 | HQ912446 | HQ912275 | **-** |
| *Neofragilaria cf nicobarica* | HK375 | KF701595 | KF701604 | KF701613 | **KT192080** |
| *Neofragilaria nicobarica* Desikachary, Prasad, & Prema | s0371 | AB433340 | **KR048216** | **KR048228** | AB425084 |
| *Neofragilaria stilus* Krzywda, Witkowski & Li | SZCZM116 | **KR048193** | **KR048213** | **KR048225** | **KR048202** |
| *Nitzschia dubiiformis* Hustedt | s0311 | AB430616 | AB430696 | **-** | **-** |
| *Nitzschia filiformis* (W. Smith) Hustedt | UTEX FD267 | HQ912589 | HQ912453 | HQ912282 | **-** |
| *Odontella aurita* (Lyngbye) C.Agardh | [ECT3788](http://www.ncbi.nlm.nih.gov/nuccore/HQ912372.1) | HQ912686 | HQ912550 | HQ912372 | **-** |
| *Odontella longicruris var. hyalina*  var. *hyalina*(J.L.B.Schröder) Cupp | [ECT3920Odhya](http://www.ncbi.nlm.nih.gov/nuccore/KC309659.1) | KC309513 | KC309587 | KC309659 | **-** |
| *Odontella sinensis* (Greville) Grunow | [CCMP1815](http://www.ncbi.nlm.nih.gov/nuccore/HQ912428.1) | HQ912564 | HQ912428 | HQ912258 | **-** |
| *Opephora sp.* Petit | s0263 | **KR048196** | **KR048218** | **KR048230** | **-** |
| *Opephora sp.* Petit | s0357 | AB430604 | AB430683 | **-** | **-** |
| *Opephora sp.* Petit | ECT3831-Opep | JN975249 | JN975264 | JN975278 | **-** |
| *Orizaformis holarctica* Witkowski, Li & Ashworth | CCMP143 | DQ514845 | DQ514763 | DQ514681 | DQ512392 |
| *Orizaformis holarctica* Witkowski, Li & Ashworth | SZCZCH111 | **KR048192** | **KR048212** | **KR048224** | **KR048203** |
| *Orizaformis holarctica* Witkowski, Li & Ashworth | SZCZCH756 | **KT119333** | **KT119338** | **KT119339** | **KT119335** |
| *Perideraion elongatum* R.W.Jordan, Y.Arai & Lobban | GU44AK-6Peridelong | KJ577939 | KJ577868 | KJ577868 | **-** |
| Perideraion montgomeryi Lobban, R.W.Jordan & M.P.Ashworth | UT HK#246 | HM627332 | HM627329 | HM627326 | **-** |
| [*Phaeodactylum tricornutum*](http://www.ncbi.nlm.nih.gov/nuccore/HQ912556.1) (Brébisson) W.Smith | CCMP2561 | HQ912556 | HQ912420 | HQ912250 | **-** |
| *Pinnularia brebissonii* (Kützing) | UTEX FD274 | HQ912604 | HQ912468 | HQ912297 | **-** |
| *Pinnularia termitina* (Ehrenberg) R.M. Patrick | UTEX FD484 | HQ912601 | HQ912465 | HQ912294 | **-** |
| [*Placoneis elginensis*](http://www.ncbi.nlm.nih.gov/nuccore/HQ912607.1) (Gregory) E.J.Cox | UTEX FD416 | HQ912607 | HQ912471 | HQ912300 | **-** |
| *Plagiogramma sp.* | SZCZCH437 | **KR048188** | **KR048206** | **KR048221** | **KR048199** |
| *Plagiogramma sp.* | HK374 | KF701594 | KF701603 | KF701612 |  |
| *Plagiogramma sp.* | HK410 | KJ577867 | KJ577904 | KJ577938 | **KT192083** |
| *Plagiogramma sp.* | HK324 | JX413546 | JX413563 | JX413580 | **KT192077** |
| *Plagiogramma staurophorum* (W.Gregory) Heiberg | HK212 | HQ912656 | HQ912520 | HQ912349 | **KT192074** |
| *Plagiostriata sp.* S.Sato & L.K.Medlin | s0388 | **KR048198** | **KR048220** | **KR048232** | AB430644 |
| *Pleurosira laevis* (Ehrenberg) Compère | UTEX FD482 | HQ912585 | HQ912449 | HQ912278 | **-** |
| *Podocystis spathulata* (Shadbolt) Van Heurck | [ECT3733-Podocystis](http://www.ncbi.nlm.nih.gov/nuccore/HQ912661.1) | HQ912661 | HQ912525 | HQ912354 | - |
| *Psammodictyon constrictum* (Gregory) D.G. Mann | s0309 | AB430617 | AB430697 | **-** | **-** |
| *Psammogramma vigoensis* S.Sato & L.K.Medlin | s0391 | **KR048194** | **KR048215** | **KR048227** | AB425085 |
| *Psammoneis japonica* S.Sato, Kooistra & L.K.Medlin | HK299 | JN975250 | JN975265 | JN975279 | **KT192076** |
| *Psammoneis obaidii* Ashworth & Sabir | UTKSA0057 | **KR059023** | **KR059022** | **KR059024** | **KR059025** |
| *Psammoneis sp.* | SZCZP70 | **KR048191** | **KR048211** | **KT119341** | **-** |
| *Pseudostriatella sp.* S.Sato, Mann & L.K.Medlin | s0384 | **KR048197** | **KR048219** | **KR048231** | **-** |
| *Rhaphoneis amphiceros* (Ehrenberg) Ehrenberg | HK373 | KF701593 | KF701602 | KF701611 | **-** |
| *Rhizosolenia imbricata* Brightwell | Har-1Rhizo | KC309543 | KC309615 | KC309686 | **-** |
| *Rhizosolenia setigera* Brightwell | CCMP1820 | HQ912561 | HQ912425 | HQ912255 | **-** |
| *Rhizosolenia setigera* Ehrenberg | ECT3845Rhizoset | KC309544 | KC309616 | KC309687 | **-** |
| *Rhopalodia contorta* Hustedt | L1299 | HQ912406 | HQ912392 | HQ912378 | **-** |
| *Rhopalodia gibba* (Ehrenberg) O. Müller | CH155 | HQ912407 | HQ912393 | HQ912379 | **-** |
| *Rhopalodia*  sp. | 9vi08.1F.2 | HQ912405 | HQ912391 | HQ912296 | **-** |
| *Rossia* sp. | E3333 | EF151968 | EF143281 | **-** | **-** |
| *Scoliopleura peisonis*Grunow | UTEX FD13 | HQ912609 | HQ912473 | HQ912302 | **-** |
| *Stauroneis acuta*W. Smith | UTEX FD51 | HQ912579 | HQ912443 | HQ912272 | **-** |
| *Staurosira construens* Ehrenberg | [UTEX FD232](http://www.ncbi.nlm.nih.gov/nuccore/HQ912587.1) | HQ912587 | HQ912451 | HQ912280 | **-** |
| *Staurosirella pinnata* (Ehrenberg) D.M.Williams & Round | CCMP330 | HQ912620 | HQ912484 | HQ912313 | **-** |
| *Stenopterobia curvula* (W. Smith) Krammer | L541 | HQ912416 | HQ912402 | HQ912388 | **-** |
| *Striatella unipunctata* (Lyngbye) C.Agardh | ECT3648-Striatella | HQ912643 | HQ912507 | HQ912336 | **-** |
| *Surirella minuta* Brébisson | UTEX FD320 | HQ912658 | HQ912522 | HQ912351 | **-** |
| *Surirella splendida* (Ehrenberg) Kützing | 19C | HQ912415 | HQ912401 | HQ912387 | **-** |
| *Synedra hyperborea* [Grunow](https://www.google.pl/url?sa=t&rct=j&q=&esrc=s&source=web&cd=1&cad=rja&uact=8&ved=0CCAQFjAA&url=http%3A%2F%2Fwww.algaebase.org%2Fsearch%2Fspecies%2Fdetail%2F%3Fspecies_id%3D34476&ei=B0fbVMvZFo3PaOyhgvAH&usg=AFQjCNGfUBWuwJxY1Yu9xwN-PDT8WB5W0Q&sig2=bNUnISr4dmAAHHSBDZI-3g&bvm=bv.85761416,d.bGQ) | [CCMP1423](http://www.ncbi.nlm.nih.gov/nuccore/HQ912621.1) | HQ912621 | HQ912485 | HQ912314 | **-** |
| *Synedra ulna* (Nitzsch) Ehrenberg | UTEX FD404 | HQ912590 | HQ912454 | HQ912283 | **-** |
| *Synedropsis cf. recta* | CCMP1620 | HQ912616 | HQ912480 | HQ912309 | **-** |
| *Tabellaria flocculosa* (Roth) Kützing | [UTEX FD133](http://www.ncbi.nlm.nih.gov/nuccore/HQ912584.1) | HQ912584 | HQ912448 | HQ912277 | **-** |
| *Tabularia cf. tabulata* | [CCMP846](http://www.ncbi.nlm.nih.gov/nuccore/HQ912615.1) | HQ912615 | HQ912479 | HQ912308 | **-** |
| *Talaroneis posidoniae* W.H.C.F.Kooistra & M.De Stefano | WK59 | AY216905 | **KR048214** | **KR048226** | AB425086 |
| *Triceratium dictyotum* P.A.Sims & R.Ross | Pan1Tricer | JX413554 | JX413571 | JX413588 | **-** |
| *Triceratium dubium* Brightwell | [CCMP147](http://www.ncbi.nlm.nih.gov/nuccore/HQ912572.1) | HQ912572 | HQ912436 |  | **-** |
| *Triceratium pentacrinus* (Ehrenberg) Wallich | Coz1Amphipentas | KJ577843 | KJ577878 | KJ577914 | **-** |
| [*Tryblionella apiculata*](http://www.ncbi.nlm.nih.gov/nuccore/HQ912600.1) Gregory | UTEX FD465 | HQ912600 | HQ912464 | HQ912293 | **-** |
