# Supplementary figures and images for: New Insights into Plagiogrammaceae (Bacillariophyta) Based on Multigene Phylogenies and Morphological Characteristics with the Description of a New Genus and Three New Species
Source: PLoS One. 2015 Oct 14;10(10):e0139300. doi: 10.1371/journal.pone.0139300 (PMC4605507; doi:10.1371/journal.pone.0139300)

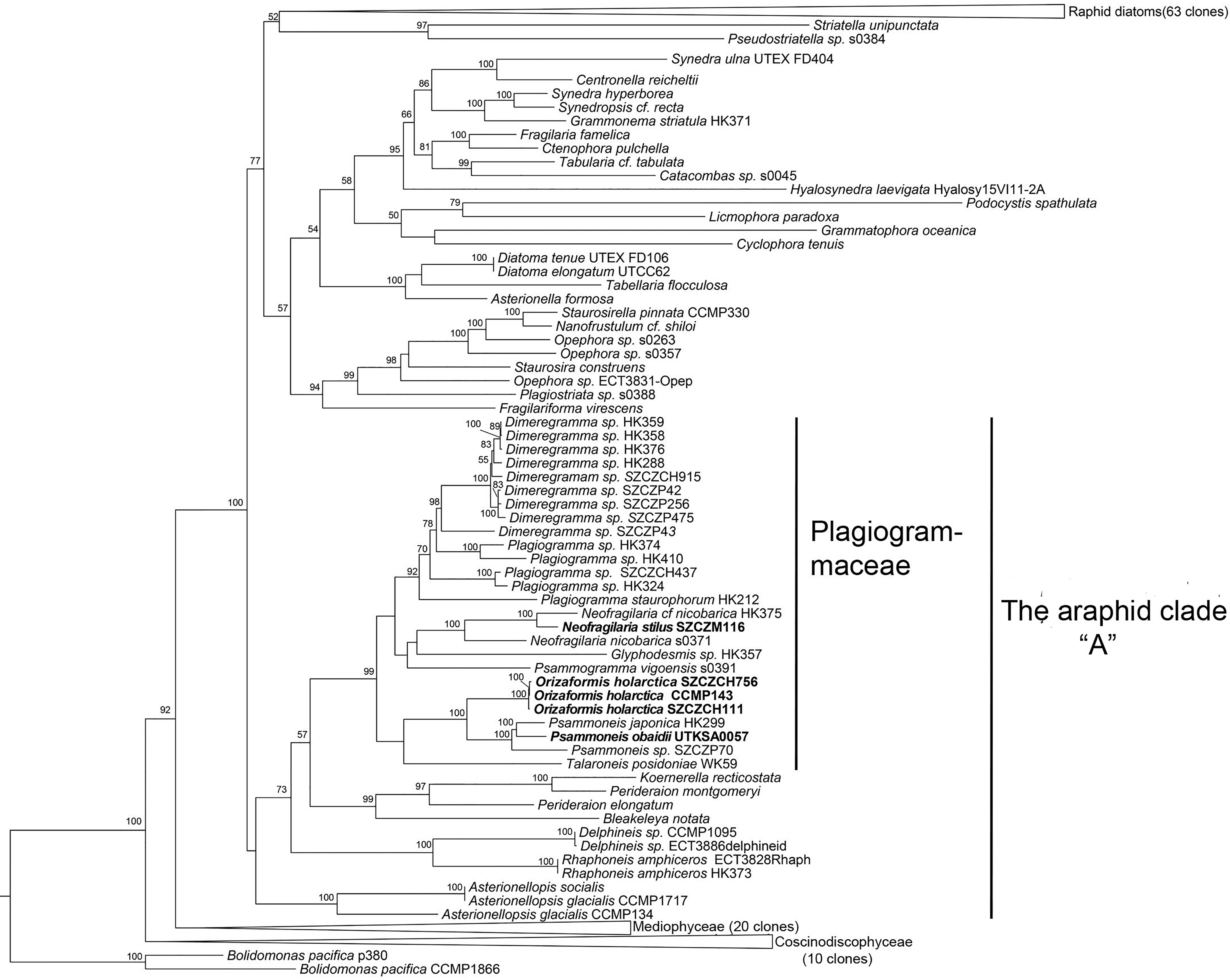

Supplement: S1 Fig — Maximum likelihood phylogeny of 161 diatoms (with bootstrap values at nodes) inferred from a concatenated alignment of SSU, rbcL and psbC markers, which constrained Dimeregramma and Plagiogramma both as a separate, monophyletic clade. The bold taxa are newly-described species. Support values lower than 50% were not included in the tree. Two Bolidomonas pacifica strains were used as outgroups. (TIF) [file pone.0139300.s002.tif]

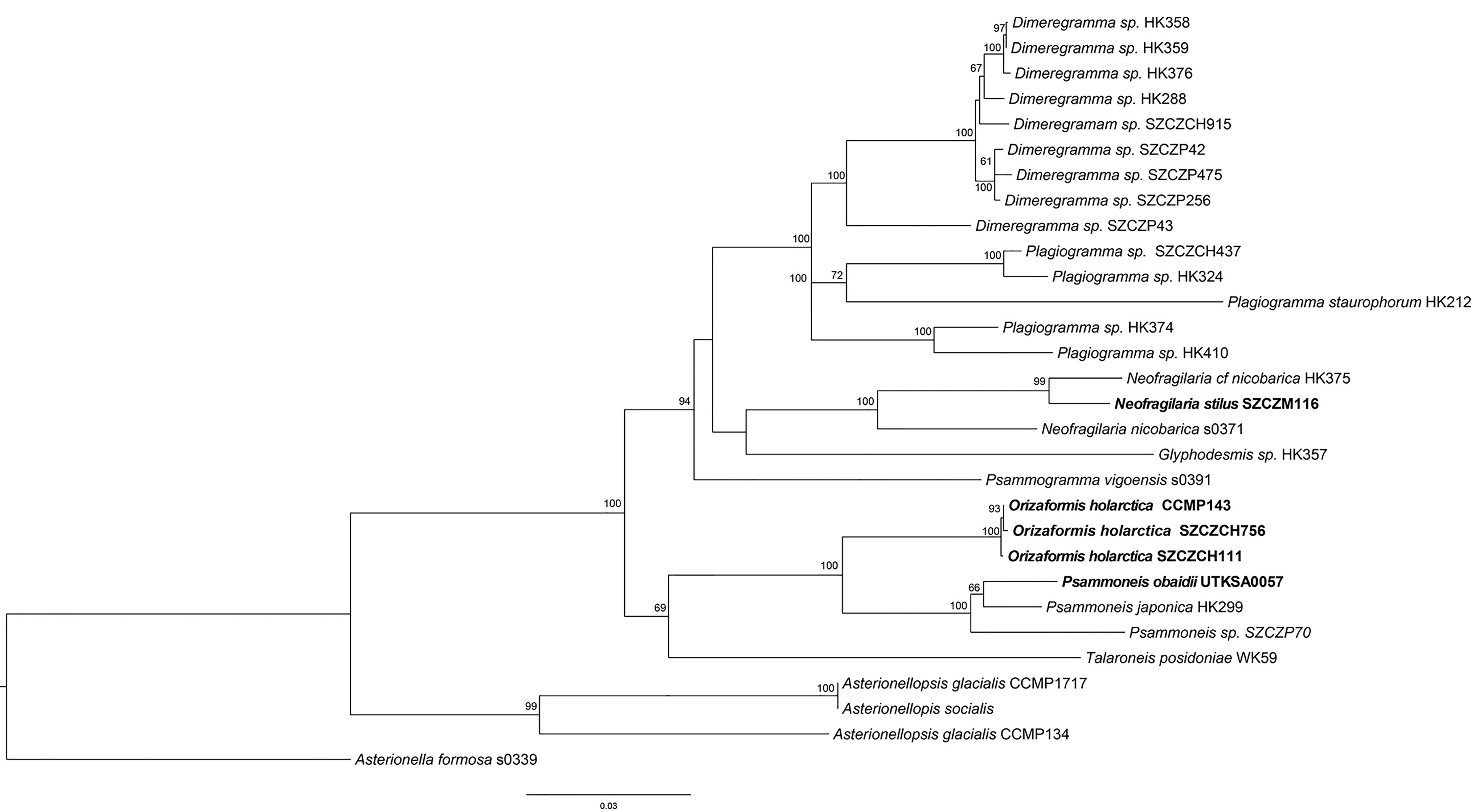

Supplement: S2 Fig — Constrained maximum likelihood tree consisting of 26 strains of Plagiogrammacean diatoms (with bootstrap values at nodes) inferred from four markers (LSU and SSU rDNA, rbcL and psbC), forcing Dimeregramma and Plagiogramma into monophyly. The bold taxa are newly-described species. Support values lower than 50% were not included in the tree. The araphid diatom Asterionella formosa was used as the outgroup. (TIF) [file pone.0139300.s003.tif]

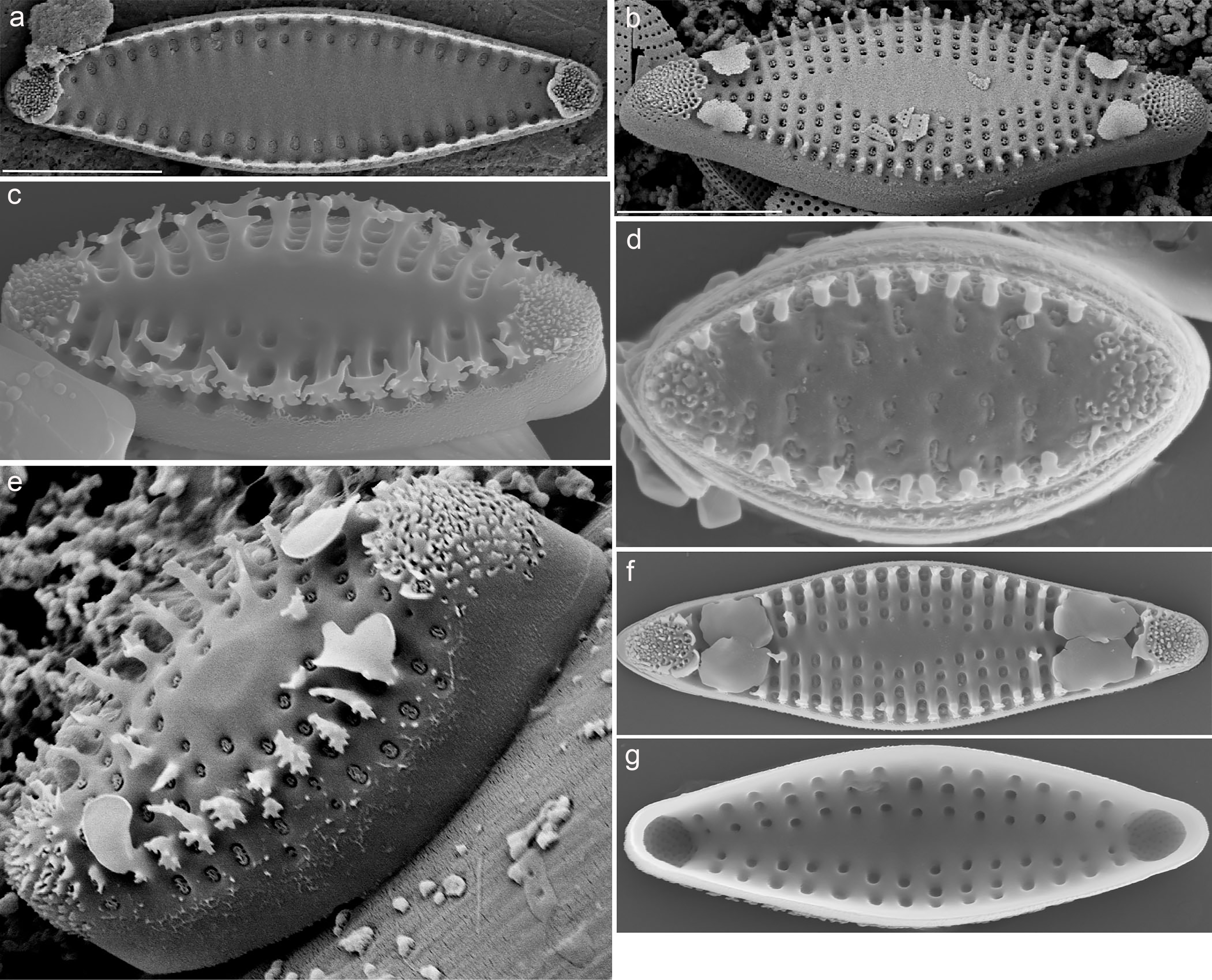

Supplement: S3 Fig — (a-b) Dimeregramma sp. (material of Malcom Giffen, slide No. 628). (c) Dimeregramma sp. HK358. (d) Dimeregramma sp. HK376. (e) Dimeregramma sp. SZCZP475. (f) Dimeregramma sp. HK288. (g) Dimeregramma sp. SZCZCH915. (TIF) [file pone.0139300.s004.tif]

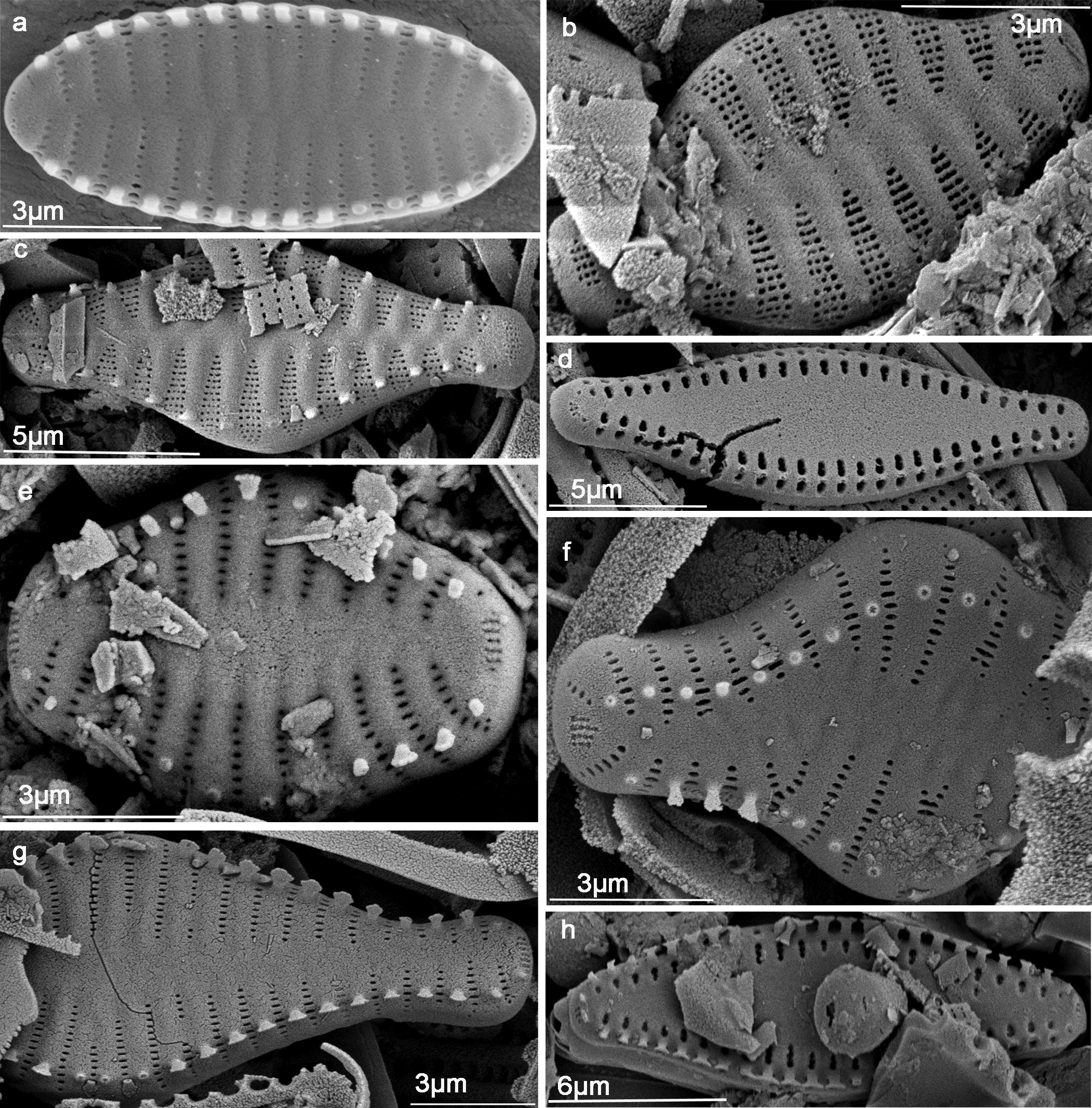

Supplement: S4 Fig — (a) Staurosira sp., natural material of SZCZ7386. (b) Punctastriata sp., natural material of SZCZ504. (c) Punctastriata sp., natural material of SZCZ503. (d) Pseudostaurosira sp., natural material of SZCZ7113. (e) Staurosira venter, natural material of SZCZ7113. (f) Staurosira construens, natural material of SZCZ5007. (g) Staurosira sp., natural material of SZCZ7113. (h) Pseudostaurosira sp., natural material of SZCZ7113. (TIF) [file pone.0139300.s005.tif]
